# Immunogenicity of prime-boost protein subunit vaccine strategies against SARS-CoV-2 in mice and macaques

**DOI:** 10.1038/s41467-021-21665-8

**Published:** 2021-03-03

**Authors:** Hyon-Xhi Tan, Jennifer A. Juno, Wen Shi Lee, Isaac Barber-Axthelm, Hannah G. Kelly, Kathleen M. Wragg, Robyn Esterbauer, Thakshila Amarasena, Francesca L. Mordant, Kanta Subbarao, Stephen J. Kent, Adam K. Wheatley

**Affiliations:** 1 Department of Microbiology and Immunology, University of Melbourne, at The Peter Doherty Institute for Infection and Immunity, Melbourne, VIC Australia; 2 Australian Research Council Centre for Excellence in Convergent Bio-Nano Science and Technology, University of Melbourne, Melbourne, VIC Australia; 3 WHO Collaborating Centre for Reference and Research on Influenza, Peter Doherty Institute for Infection and Immunity, Melbourne, VIC Australia; 4 Melbourne Sexual Health Centre and Department of Infectious Diseases, Alfred Hospital and Central Clinical School, Monash University, Melbourne, VIC Australia

**Keywords:** Cellular immunity, Humoral immunity, Vaccines, SARS-CoV-2

## Abstract

SARS-CoV-2 vaccines are advancing into human clinical trials, with emphasis on eliciting high titres of neutralising antibodies against the viral spike (S). However, the merits of broadly targeting S versus focusing antibody onto the smaller receptor binding domain (RBD) are unclear. Here we assess prototypic S and RBD subunit vaccines in homologous or heterologous prime-boost regimens in mice and non-human primates. We find S is highly immunogenic in mice, while the comparatively poor immunogenicity of RBD is associated with limiting germinal centre and T follicular helper cell activity. Boosting S-primed mice with either S or RBD significantly augments neutralising titres, with RBD-focussing driving moderate improvement in serum neutralisation. In contrast, both S and RBD vaccines are comparably immunogenic in macaques, eliciting serological neutralising activity that generally exceed levels in convalescent humans. These studies confirm recombinant S proteins as promising vaccine candidates and highlight multiple pathways to achieving potent serological neutralisation.

## Abstract (web summary)

Current vaccine strategies for SARS-CoV-2 focus on eliciting neutralising antibodies to the spike protein (S), but differences in immunogenicity of full-length S versus receptor binding domain (RBD) only aren’t fully understood. Here, the authors show immunogenicity of different prime-boost strategies with S and/or RBD in mice and macaques.

## Introduction

The rapid onset and global spread of the SARS-CoV-2 pandemic have spurred unprecedented global scientific efforts to develop, test and manufacture novel protective vaccines. The spike (S) glycoprotein of SARS-CoV-2 is a clear target for vaccines designed to elicit neutralising antibodies to prevent infection. Recent studies suggest neutralising antibodies can protect macaques against SARS-CoV-2^1–3^ and observational human studies also suggest neutralising antibody responses are protective against re-infection^4^. S is a type 1 viral fusion protein, expressed as a single polypeptide and cleaved into S1 and S2 subunits, with a heterotrimeric quaternary structure common to many respiratory viruses reviewed in^5^. Cell entry is mediated by engagement of the enzyme ACE2 on the target cell surface by the viral receptor-binding domain (RBD), localised within the C-terminal domain of S1^6,7^. Antibodies capable of preventing RBD binding to ACE2 can therefore prevent infection and constitute an efficient pathway to neutralisation.

Viruses employ numerous strategies to avoid immune recognition of viral entry proteins, including heavy decoration with N-linked glycans^8^, employing immune distraction or escape by focussing host immunity onto highly mutable regions^9^. A shared challenge for vaccine development against viral glycoproteins is therefore ensuring maximum B-cell recognition of neutralising epitopes critical for viral replication (on-target), while minimising responses to poorly conserved epitopes or those with no antiviral capacity (off-target).

Currently, several S-based vaccines are entering early stage clinical trials, including recombinant trimeric S proteins, monomeric or trimeric RBD domains, and analogues delivered by viral vectors or mRNA^10–15^. However, the relative merits of these immunogens are currently unclear. In particular, does the use of a smaller vaccine target, such as the RBD, drive a more focussed neutralising antibody response? Or alternatively, do additional epitopes across the larger S protein make additive contributions to immunogenicity or neutralisation that counteract any off-target immune distraction? Here we directly compare the immunogenic profile of SARS-CoV-2 S and RBD immunogens in mice and non-human primates using various prime-boost approaches (Fig. S1). We find in mice that RBD is relatively poorly immunogenic compared to S, with primary immunisation compromised by a reduced capacity to efficiently induce germinal centre B cells and recruit effective T follicular helper cells. In contrast, immunisation with S alone, or boosting S-primed animals with S or RBD, is potently immunogenic, reliably eliciting strong binding and neutralising titres in immunised mice. In more genetically diverse non-human primates, two immunisations with either S or RBD immunogens were comparably immunogenic and produced strong serological neutralising responses. Overall, we find that immunisation with recombinant S immunogens reliably elicits potentially protective humoral immunity at levels in excess of those observed in convalescent humans.

## Results

### SARS-CoV-2 spike but not RBD is potently immunogenic in mice

**Fig. 1:**
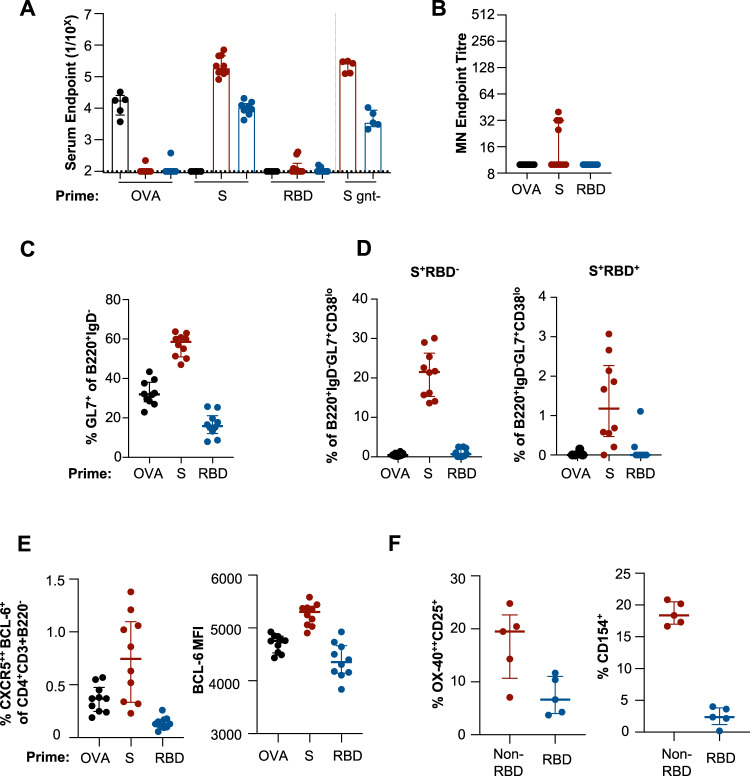
Primary immunogenicity of SARS-CoV-2 subunit proteins in C57BL/6 mice. Mice were immunised intramuscularly with S, RBD or OVA proteins and immune responses assessed 14 days post immunisation (*n* = 10 animals across two independent experiments, S gnt- *n* = 5). **A** Reciprocal serum endpoint dilutions of S- (red; *n* = 10), RBD- (blue; *n* = 10) or OVA-specific IgG (black; *n* = 5) were measured by ELISA. Dotted lines denote the detection cut off (1:100 dilution). **B** Serum neutralisation activity was assessed using a microneutralisation assay. **C** Draining lymph node germinal centre activity assessed by GL7 expression in B220^+^IgD^−^ B cells. **D** Frequency of germinal centre B cells (B220^+^IgD^−^GL7^+^CD38^lo^) specific for Spike (S^+^RBD^−^) or RBD (S^+^RBD^+^) probes. **E** Frequency of TFH cells (CXCR5^++^BCL-6^+^CD4^+^CD3^+^B220^−^) and corresponding median fluorescent intensity of BCL-6. **F** Antigen-specific TFH cells were identified as either OX-40^++^CD25^+^ or CD154^+^ following 18 h of stimulation with S (minus RBD) or RBD peptide pools (*n* = 5 animals). Antigen-specific responses are presented after background subtraction using a DMSO control. Data is presented as median ± IQR. Source data are provided as a source data file.

The primary immunogenicity of SARS-CoV-2 S and RBD was assessed in groups of C57BL/6 mice vaccinated with S, RBD or control ovalbumin (OVA) proteins. A single immunisation of S formulated with Addavax (an MF-59-like squalene adjuvant) was highly immunogenic, eliciting high reciprocal serum endpoint titres of S-specific antibody at day 14 post immunisation (Fig. 1A; median 1.85 × 10^5^; IQR 1.34–4.61), but without inducing significant neutralisation activity (Fig. 1B). In contrast, a single immunisation of RBD elicited minimal serum antibody in line with other reports of sub-optimal immunogenicity of RBD in mice^16^ and recent Phase I trials comparing RBD or S encoded by RNA-based vaccines^15^. Comparable serological titres were observed if measured at day 28 post immunisation (Fig. S2), or if SARS-CoV-2 immunogens were formulated with an alternative MPLA-based liposomal adjuvant (Fig. S3A). Although S is extensively glycosylated^8^, limiting complex glycan deposition by expression in HEK293 cell lines lacking N-acetylglucosaminyltransferase I (S gnt-) did not negatively impact the potent immunogenicity of S (Fig. 1A).

The elicitation of SARS-CoV-2 specific B and T cell responses in the draining iliac and inguinal lymph nodes (LN) was assessed by flow cytometry. S-immunised animals displayed robust induction of germinal centre (GC) B cells measured by intracellular surface GL7^+^ (median 58.5%; IQR 51.0–63.8) expression (Fig. 1C; gating in Fig. S4). Immunisation with OVA-induced intermediate frequencies of GL7^+^ (median 32.0%; IQR 28.2–38.1) B cells, with minimal detection of GC formation in RBD-immunised animals (GL7^+^; median 15.9%; IQR 12.1–21.1). The specificity of GC B cells (IgD-B220^+^GL7^+^CD38^lo^) was examined using recombinant S and RBD probes^17^. Both S-specific (S^+^RBD^−^) and RBD-specific (S^+^RBD^+^) were reliably detected in S-immunised animals, constituting 21.5% (IQR 15.3–26.3) and 1.2% (IQR 0.47–2.27) of GC B cells, respectively (Fig. 1D). Mirroring the serum antibody response, few S- or RBD-specific GC B cells were observed in RBD-immunised animals.

Consistent with GC B-cell frequencies, immunisation with S induced high frequencies of T follicular helper (TFH) cells (CXCR5^++^BCL-6^+^; median 0.7%; 0.3–1.1) relative to OVA (median 0.4%; 0.2–0.8) or RBD (median 0.13%; 0.1–0.2) (Fig. 1E; gating Fig. S2). The median fluorescent intensity (MFI) of BCL-6 expression in TFH was notably higher in S-vaccinated animals (median 5303 for S, 4756 for OVA and 4353 for RBD) (Fig. 1E). Analysis of TFH specificity using an activation-induced marker (AIM) assay^18^ and pools of RBD or non-RBD S overlapping peptides indicated that the TFH response is dominated by non-RBD-localised epitopes in S-immunised animals (Fig. 1F). Further, RBD peptides elicited weak CD154 responses upon re-stimulation in comparison to non-RBD peptides among both TFH (Fig. 1F) and CXCR5^−^ CD4 T cells (Fig. S6A). Epitope mapping of peptides spanning the RBD indicated that only three RBD-derived peptides were recognised by CD4 T cells in C57BL/6 mice (Fig. S6B, C), one of which was substantially more immunogenic than the others (P99, sequence TNVYADSFVIRGDEV). In contrast, a more extensive number (>8) of S epitopes were identified outside the RBD (four of which are shown in Fig. S6B, C). In S-vaccinated animals, the three RBD-derived epitopes were subdominant to the most immunogenic non-RBD S epitopes and relatively poor inducers of CD154 expression (Fig. S6B, C). These data suggest that the presence of the single relatively immunogenic TFH epitope (P99) within the SARS-CoV-2 RBD is insufficient to drive a robust CD4 T cell response in comparison to the greater number of immunodominant epitopes available in full-length S.

The primary immunogenicity of the RBD was similarly muted in BALB/c mice in comparison to S, with poor induction of RBD-directed antibody, GC B cells and TFH responses (Fig. S7). Overall, we find that S is potently immunogenic in both mouse strains, with GC B cell and TFH responses largely targeted at regions outside the RBD. In contrast, the immunogenicity of the RBD is constrained in mice, likely in part through suboptimal recruitment of quality TFH responses.

### Homologous spike and heterologous spike/RBD prime-boost immunisations elicit potent binding and neutralising antibody responses in mice

**Fig. 2:**
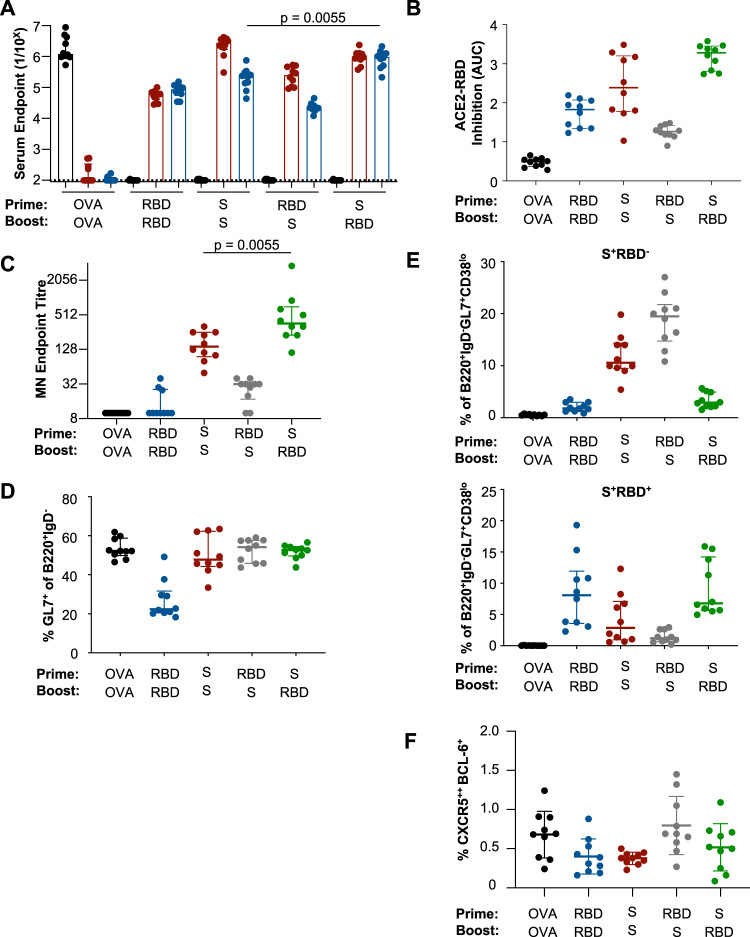
Prime-boost immunisation of SARS-CoV-2 subunit proteins in C57BL/6 mice. Mice were serially immunised intramuscularly at a 21-day interval with S, RBD or OVA proteins and immune responses assessed 14 days post boost (*n* = 10 animals across two independent experiments). **A** Reciprocal serum endpoint dilutions of S- (red), RBD- (blue) or OVA-specific IgG (black) were measured by ELISA. Dotted lines denote the detection cut off (1:100 dilution). *P* values were derived by two-tailed Mann–Whitney U tests. **B** The capacity of serum antibodies to inhibit the interaction of RBD and human ACE2 was assessed by ELISA. **C** Neutralisation activity in the serum was assessed using a microneutralisation assay. **D** Draining lymph node germinal centre activity assessed by GL7 expression in B220^+^IgD^−^ B cells. **E** Frequency of germinal centre B cells (B220^+^IgD^−^GL7^+^CD38^lo^) specific for spike (S^+^RBD^−^) or RBD (S^+^RBD^+^) probes. **F** Frequencies of TFH cells (CXCR5^++^BCL-6^+^CD4^+^CD3^+^B220^−^). *P* values were derived by two-tailed Mann–Whitney U tests. Data are presented as median ± IQR. Source data are provided as a source data file.

Despite potentially compromised immunogenicity, the small antigenic target of the RBD remains attractive for focusing immunity upon protective neutralising epitopes. However, the recent identification of the N-terminal domain (NTD)^19^ and other alternative S-localised protective epitopes^20,21^ highlights additional antibody targets for vaccine protection. To assess the relative merits of RBD-focussed antibody responses versus more holistically targeting the entire S, we primed C57BL/6 mice with S or RBD, and then boosted 21 days later with either homologous or heterologous immunogens. Homologous S prime-boost (S-S) elicited high reciprocal serum endpoint titres of both S- (median 2.77 × 10^6^; IQR 1.71–3.62) and RBD-specific antibodies (2.28 × 10^6^; IQR 1.23–3.17) (Fig. 2A). In contrast to a single RBD dose, we found that homologous RBD prime-boost (R-R) immunisation was capable of eliciting modest serum titres of S- and RBD-specific antibodies, suggestive of memory B cells elicited after the RBD-prime. RBD prime-S boost (R-S) induced similarly modest titres. However, we find RBD boosting of S-primed animals (S-R) markedly increased RBD-specific serum antibody titres 4.2-fold relative to S-S (*p* = 0.0055). This was also evidently using a blocking ELISA approach, where S-R immunised animals displayed a markedly higher proportion of RBD-specific antibodies compared to S-S immunised animals (Fig. S9). Inhibition of ACE2-RBD interaction by serum antibodies was similarly enhanced in the S-R group compared to the S-S group (Figs. 2B and  S8). Selective RBD focussing translated into increased serum neutralisation, with S-R eliciting 2.5-fold higher activity (median 361; IQR 226–706) compared to S-S (143; IQR 96–254) (*p* = 0.0055) (Fig. 2C). Neither R-R nor R-S immunisation reliably induced significant serum neutralising activity, although they elicited modest levels of antibodies that block ACE2-RBD engagement. The capacity of S-S and S-R immunisation to elicit potent binding and neutralising responses in C57BL/6 mice was mirrored in BALB/c mice (Fig. S10).

The profile of B and T cell immunity was assessed in draining lymph nodes 2 weeks after the boost immunisation. R-R elicited the lowest GC B-cell responses measured by surface GL7^+^ expression (22.3%; IQR 20.5–31.6) (Fig. 2D). Both S-S (GL7^+^: 47.8%, IQR 44.3–62.3) and S-R (GL7^+^: 52.7%; IQR 49.7–54.5) displayed intermediate induction of GC activity, while R-S immunised animals showed robust GC induction (GL7^+^: 54.2%; IQR 45.9–57.6), likely reflecting a primary response against non-RBD epitopes within S. The hierarchy of GC activity was mirrored in the frequency of probe-specific GC B cells, with R-S (19.5% (IQR:14.7–21.8) and S-S (10.6%; IQR 9.46–14.4) eliciting high frequencies of S-specific GC B cells (Fig. 2E), while low frequencies were observed for R-R (1.83%; IQR 1.25–2.98) and S-R groups (2.9%; IQR 2.21–4.96). RBD-specific GC B cells were highest in R-R (8.07%; IQR 3.58–11.9) and S-R (6.79%; IQR 5.65–14.2) groups, with lower frequencies after S-S (2.86%; 0.96–7.11%) and R-S regimens (1.20%; IQR 0.67–2.62). GC TFH frequencies were highest in R-S animals (0.69%, IQR 0.6–1.1) compared to the other groups (R-R 0.4%, IQR 0.2–0.6; S-S 0.4%, IQR 0.3–0.4%; S-R 0.5%, IQR 0.2–0.7) (Fig. 2F). The distribution of GC B and TFH responses after prime-boost immunisation were consistent in BALB/c mice (Fig. S10).

### Both RBD and S immunogens elicit potent antibody responses and serum neutralising activity in non-human primates

**Fig. 3:**
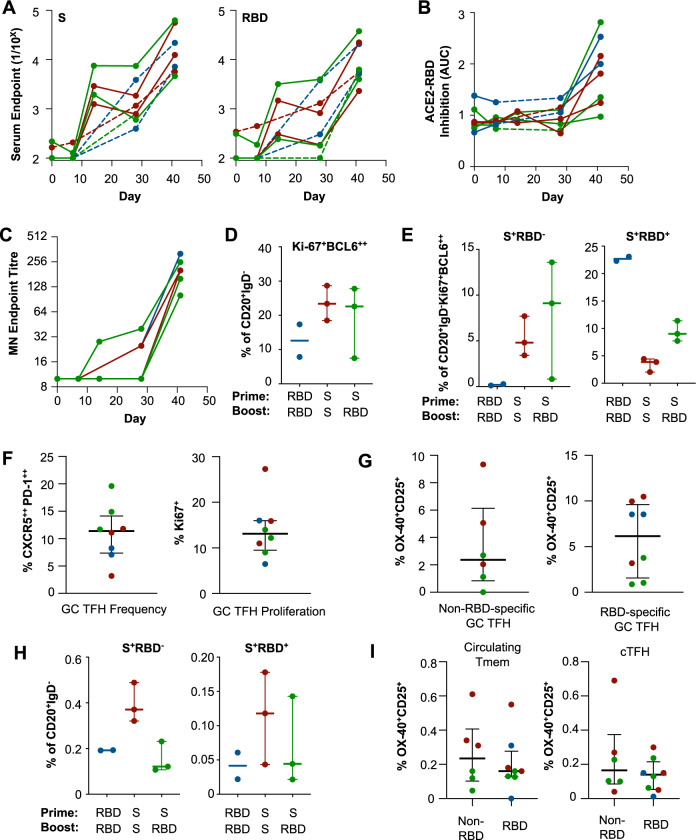
SARS-CoV-2 spike and RBD immunogens elicit robust B and TFH responses in macaques. Macaques were serially immunised intramuscularly at a 28-day interval with S or RBD immunogens and immune responses assessed 13–14 days post boost (R-R in blue (*n* = 2), S-S in red (*n* = 3) and S-R in green (*n* = 3)). Dashed lines indicate animals without day 14 post prime sampling. **A** Reciprocal endpoint titres of S- or RBD-specific IgG were measured in longitudinal plasma samples by ELISA. **B** The capacity of plasma antibodies to inhibit the interaction of RBD and human ACE2 was assessed by ELISA. **C** Neutralisation activity in plasma was assessed using a microneutralisation assay. **D** Germinal centre CD20^+^IgD^−^ B cells were quantified based on Ki-67^+^BCL-6^+^ expression and frequencies of (**E**) spike- (S^+^RBD^−^) or RBD-specific (S^+^RBD^+^) populations determined by flow cytometry. **F** Frequency of germinal centre TFH cells (CD3^+^CD4^+^CXCR5^++^PD-1^++^) and Ki-67^+^ expression in draining lymph nodes. **G** Antigen-specific TFH cells identified by OX-40^+^CD25^+^ upregulation following 18 h of stimulation with S (minus RBD) or RBD peptide pools. **H** Frequency of circulating memory B cells within PBMC specific for spike (S^+^RBD^−^) or RBD (S^+^RBD^+^). **I** Antigen-specific circulating memory CD4 T cells (Tmem; CD3^+^CD4^+^CD95^+^CXCR5^−^) or circulating TFH cells (cTFH; CD3^+^CD4^+^CD95^+^CXCR5^+^) identified as OX-40^+^CD25^+^ following 18 h of stimulation with S (minus RBD) or RBD peptide pools. Antigen-specific T cell responses (**G**, **I**) are presented after background subtraction using a DMSO control. Data is presented as median ± IQR. Source data are provided as a source data file.

To enable serial measurements in a highly relevant animal model, we next immunised pig-tailed macaques (*Macaca nemestrina*) with S and RBD protein vaccines formulated with an MPLA liposomal adjuvant^22^. Two doses in all three vaccine regimens, R-R (*n* = 2), S-S (*n* = 3) and S-R (*n* = 3), reliably elicited robust serum antibody responses against S and RBD proteins (Fig. 3A), together with a corresponding rise in both ACE2-RBD inhibitions (Fig. 3B) and neutralising activity (Fig. 3C; median titre 202, IQR 160–241). Responses in macaques were notably more variable than mice. Nevertheless, we did find that heterologous prime-boost immunisation in the S-R animals was reliably associated with focusing of the anti-S antibody response toward the RBD and away from the N-terminal domain (NTD) of S1 (Fig. S9).

B and T cells responses to immunisation were assessed 2 weeks after the boost in the draining lymph nodes of immunised animals, identified based upon staining with a co-formulated tracking dye^23^. While 7/8 animals exhibited dye staining of the iliac LN, the primary site of antigen drainage from quadricep intramuscular vaccination^23^, NM224 showed dye staining of only the inguinal LN. Total GC B and TFH frequencies, as well as antigen-specific GC B and TFH frequencies, were enriched in the dyed inguinal LN compared to the unstained iliac LN^23^.

Robust GC B-cell responses (Fig. 3D) (CD20^+^IgD^−^Ki-67^+^BCL-6^+^; gating Fig. S11A) were elicited in all animals, with S-S (23.4%, range 18.5–28.7) and S-R (22.6%, range 7.5–27.8) immunised animals displaying higher GC frequencies relative to R-R (12.6%, range 7.86–17.4) immunised animals. GC B-cell specificity was also assessed by S- or RBD-probe binding (Fig. 3E). RBD-specific B cells were detected in all groups, with R-R immunised animals displaying the highest levels (22.7%, range 22.3–23.1) followed by the S-R (9.02%, range 7.73–11.4) and S-S (3.88%, range 2.03–4.43) groups. In contrast, high frequencies of S-specific GC B cells were elicited in S-S (4.81%, range 3.41–7.71) and S-R (9.11%, range 0.82–13.6) immunised, but not R-R immunised animals.

GC TFH (CD3^+^CD4^+^CXCR5^++^PD-1^++^; gating in Fig. S12A) were also detected in all draining lymph nodes (11.4% of total LN CD4^+^ T cells), with a median of 13% exhibiting recent proliferation as measured by Ki-67 expression (Fig. 3F). S-specific TFH targeting either non-RBD or RBD peptides were detected in all animals; interestingly, RBD-derived peptides tended to be more frequently recognised by GC TFH than non-RBD epitopes (2.4% non-RBD, S-specific versus 6.2% RBD-specific; Fig. 3G).

The elicitation of memory lymphocyte populations is a key aim for protective vaccines. Circulating S- and RBD-specific memory B cells (CD20^+^IgD^−^; gating Fig. S11B) were assessed in PBMCs (Fig. 3H). S-specific memory B cells were highest in S-S immunised animals (0.37%, range 0.32–0.49), and approximately equivalent frequencies in R-R (0.1925%, range 0.192–0.193), and S-R (0.12%, range 0.11–0.23) immunised animals. In contrast, RBD-specific memory B cells were less frequently detected overall, with S-S (0.12%, range 0.04–0.18) animals displaying the highest level, followed by the S-R (0.04%, range 0.02–0.14) and R-R (0.04%, range 0.02–0.06) groups.

Both S-specific memory CD4 T cells (Tmem) and circulating TFH (cTFH; gating Fig. S12B, C) were detected in blood 2 weeks after the vaccine boost (Fig. 3I). Tmem responses were predominately observed within the central memory (TCM, CD95^+^CD28^+^) subset rather than the effector memory (TEM, CD95^+^CD28^lo^) population (Fig. S13A). Stimulation of PBMC with overlapping peptides spanning the S protein-induced production of IL-2, IL-5, IL-10 and IL-17A, with no major differences between vaccine regimens (Fig. S13B). In contrast to previous observations in convalescent human donors^17^, NHP S-specific cTFH recognised non-RBD and RBD-derived peptides at similar frequencies (Fig. 3J). Among all animals, RBD-specific cTFH frequencies correlated with S antibody titres suggesting that, analogous to humans^24–28^, antigen-specific cTFH constitute a useful biomarker of vaccine immunogenicity in NHP models (*p* = 0.04, Fig. S13C).

### Profile of responses in mice, macaques and humans

**Fig. 4:**
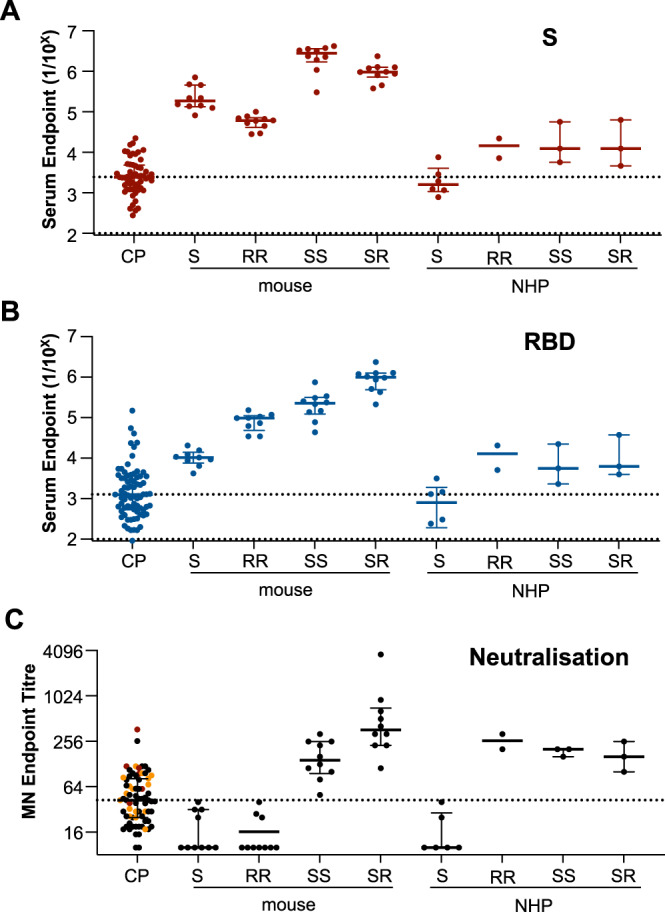
Comparative serological antibody and neutralising activity against SARS-CoV-2 across mice, macaques and convalescent humans. Serum from mice (*n* = 10 per group), and plasma from macaques (*n* = 2 for RBD/RBD group, *n* = 3 for Spike/RBD and Spike/Spike groups) and human convalescent donors (*n* = 72) were assessed for endpoint total IgG titres measured by ELISA against **A** S or **B** RBD. **C** Neutralisation activity in the plasma (human, macaques) or serum (mouse) was assessed using a microneutralisation assay. **A**–**C** Dotted lines represent median of human convalescent donors. Severity of infection for the convalescent cohort is indicated as mild (black), moderate (orange) and severe (red). Data are presented as median ± IQR. Source data are provided as a source data file.

While our results suggest that adjuvanted protein vaccines are reliably immunogenic in mice and non-human primates, it remains unclear how well these models recapitulate human immunity to SARS-CoV-2. As a reference, S- and RBD-specific antibody and serum neutralisation titres were assessed in a panel of 72 convalescent donors recovered from COVID-19 (participant details in Table 1). We find that both S-S and S-R immunised mice display high comparative titres, with potent serum neutralisation appearing upon boosting with SARS-CoV-2 immunogens (Fig. 4). Similarly, immunisation of NHP with one or two doses of SARS-CoV-2 immunogens, elicited binding antibodies at levels above the median observed in convalescent individuals. Neutralising responses in NHPs exceeded levels observed in convalescent individuals upon boosting with SARS-CoV-2 immunogens. In contrast to the highly RBD-focussed responses in immunised animals, particularly those receiving heterologous S-R prime-boost vaccines, RBD-specific antibody made up an approximate ~35% of S-specific responses in convalescent individuals, comparable to responses to the NTD (Fig. S9). Overall, these data suggest differences in both the magnitude, and qualitative aspects of the humoral response, exist between S-specific antibody elicited by infection versus by immunisation with pre-fusion stabilised S immunogens.

**Table 1:** Characteristics of COVID-19 convalescent cohort.

	Convalescent subjects (*n* = 72)
Proportion female, % (*n*)	41.6% (30)
Age, median (IQR)	56 (62, 49)
Time since positive PCR test, median (IQR)*	36 (31.5, 45)
Illness severity^, % (*n*)	
—Mild	68% (49)
—Moderate	22% (16)
—Severe	8% (6)

*Negative PCR test result for six individuals, all subjects seropositive.

^Available for 71/72 participants.

To examine the influence of host immunogenetics on antibody and B-cell responses, we sorted and sequenced S- and/or RBD-specific B cells from convalescent subjects and immunised macaques, and germinal centre B cells from immunised mice. We find vaccine-elicited GC B cells in mice are drawn primarily from VH1-like gene families, in comparison to the VH3 and VH4 families that are predominant in RBD- and S-specific B cells recovered in the LN or PBMC of an immunised macaque (Fig. S14). In contrast to immunised animals, RBD- and S-specific B cells sequenced from PBMC of convalescent subjects are drawn from a range of V-gene families, although we and others have reported biasing toward some VH3 family genes including VH3-30 and VH3-53/VH-3-66^17,29^. In terms of CDR-H3 length, recovered CDR-H3 from immunised mice were relatively short (median 11; IQR 10–12) as has been consistently reported^30^. In contrast, longer CDR-H3 were seen in macaque LN (15; IQR 13–18) and PBMC (15; IQR 13–18), which were more comparable to lengths observed for RBD- and S-specific immunoglobulin sequences observed in convalescent subjects (15; IQR 13–18).

## Discussion

Preliminary reports from SARS-CoV-2 vaccine candidates suggest both S^10–13^ and RBD^14,15^ are immunogenic in human subjects. However, the comparative performance of each immunogen in pre-clinical animal models, and the potential for combinatorial use in heterologous prime-boost strategies, is unclear. In line with other reports^15,16^, we find the intrinsic immunogenicity of the RBD is limited in mice after a single or two doses, likely reflecting inefficient recruitment of high-quality TFH in the primary response, analogous to our previous report for influenza hemagglutinin stem immunogens^31^. In contrast, priming of mice with S resulted in consistently higher serum titres and neutralising activity, irrespective of the subsequent boosting immunogen, highlighting the impact of a broader TFH repertoire in modulating the magnitude of immune responses after boosting. RBD was notably more immunogenic in vaccinated non-human primates in the context of greater MHCII loci and allelic diversity compared to mice. Nevertheless, the limited recognition of the RBD by B and cTFH cells following SARS-CoV-2 infection^17^ suggests that at a population level, RBD-based immunogens might be less reliable vaccine antigens than S, which was robustly immunogenic in all species. It should be noted however that approaches such as multimerization of RBD subunits^32,33^, or alternatively delivering RBD as mRNA instead of recombinant protein^34^, can significantly boost the immunogenicity in vivo and potential utility as a vaccine immunogen.

Selective recall of RBD responses by heterologous S-R prime-boost immunisation focussed antibody recognition onto the ACE2 recognition site in mice, as evidenced by enhanced ACE2-RBD inhibition activity and a minor increase in neutralising activity. In contrast, enhanced neutralisation activity was not seen in S-R immunised macaques, where titres were equivalent to S-S animals. This mirrors our recent findings in convalescent humans, where serological neutralising activity was not exclusively RBD-directed^17^, and suggests alternative S-localised epitopes such as the NTD^19^, may contribute to vaccine-elicited protection. However, we note that macaques immunised with S-R did display increased RBD, and correspondingly, reduced NTD reactivity within the S-specific antibody response, highlighting that heterologous immunisation can drive immune-focusing onto the RBD. Analogous approaches may potentiate the capacity to modulate the durability or potency of RBD versus NTD antibodies in humans in response to prototypic SARS-CoV-2 vaccines.

While mice have tremendous utility for immunological research including vaccine development efforts, we found divergent gene family usage and constrained CDR-H3 lengths in vaccine-elicited B-cell responses in mice compared to immunised macaques or convalescent humans. These differences might explain the comparatively high serological binding titres required in mice for neutralisation activity compared to humans and immunised primates. Notably, many reported neutralising human monoclonal antibodies specific for epitopes outside the RBD, such as the N-terminal domain^19,20^, display extended CDR-H3 loops of 20–25AA, considerably longer than what is commonly seen in mice. Therefore, genetic constraints in the murine immunoglobulin repertoire might impact vaccine-elicited humoral immunity to S, with implications for stratification of SARS-CoV-2 vaccines for eventual human use.

Notably, the differences in immunogenicity between S and RBD observed in mice were not reliably recapitulated in NHP. We have previously observed differences in vaccine antigen immunogenicity between small and large animal models^35^, highlighting the value of NHP models in clarifying the translational pathway of prototypic vaccines to humans. Multiple factors likely contribute to the differential responses between species including size, microbiome and the fact that macaques are an outbred population with diverse MHC and immunoglobulin alleles. Differences in these highly polymorphic genes could contribute to the increased recognition of RBD epitopes among B and T cells in macaques compared to mice and/or humans. Additional refinements to vaccine dose or timing may be required to further optimise the immunogenicity of these antigens in macaques. Ultimately, larger NHP studies and/or human clinical trials will be required to robustly assess differences between homologous and heterologous vaccine regimens.

Most reports of clinical and pre-clinical SARS-CoV-2 vaccine assessment have been benchmarked against convalescent subjects recovered from COVID-19. However, numerous groups including ours have reported that neutralising titres in convalescent subjects are comparatively weak^11,12,14,15,17^ and appear to wane rapidly^36–38^. Here a simple two protein regime using licensure-friendly adjuvants was able to elicit superior binding and neutralising antibody responses. Prototypic vaccines induced strong GC activity in draining lymph nodes, driving maturation of S-specific B cells, and seeded memory T and B-cell responses in the blood. Overall, our study suggests that vaccination constitutes a more robust and reliable pathway to serological protection against SARS-CoV-2 than natural infection, similar to other pathogens such as human papillomavirus^39,40^.

## Methods

### Ethics statement

Animal studies and related experimental procedures were approved by the University of Melbourne Animal Ethics Committee (no. 1714193, no. 1914874). Macaque studies and related experimental procedures were approved by the Monash University Animal Ethics Committee (no. 23997). Human clinical study protocols were approved by the University of Melbourne Human Research Ethics Committee (no. 2056689), and all associated procedures were carried out in accordance with approved guidelines. All participants provided written informed consent in accordance with the Declaration of Helsinki.

### Expression of coronavirus proteins

Recombinant SARS-CoV-2 spike and RBD proteins were expressed and validated for serological and flow cytometric assays^17^. Briefly, the ectodomain of SARS-CoV-2 (isolate WHU1; residues 1–1208) with furin cleavage site removed and P986/987 stabilisation mutations^41^, a C-terminal T4 trimerisation domain, Avitag and His-tag, was expressed in Expi293 cells and purified by Ni-NTA and size-exclusion chromatography. The SARS-CoV-2 RBD^42^ with a C-terminal His-tag (residues 319–541; kindly provided by Florian Krammer) was expressed in Expi293 cells and purified by Ni-NTA and size-exclusion chromatography. The SARS-CoV-2 NTD (residues 1–290) was synthesised with a C-terminal Avitag and His-tag, cloned into mammalian expression vectors and expressed in Expi293 cells. Recombinant NTD protein was purified using Ni-NTA and size-exclusion chromatography and verified using SDS-PAGE.

### Animal immunisations

Five micrograms of S, RBD or OVA proteins were formulated in PBS at a 1:1 ratio with Addavax adjuvant (InvivoGen) or a 1:2 ratio with Monophosphoryl Lipid A (MPLA) liposomes (Polymun)^22^. C57BL/6 or BALB/c mice were anesthetised by isoflurane inhalation prior to intramuscular injection of 50 μL vaccine in each hind quadriceps. Primary responses were assessed 14 or 28 days after prime immunisation. Booster immunisations were administered 3 weeks post prime, and responses assessed 14 or 28 days after boost.

Pigtail macaques (*Macaca nemistrina*) were housed in the Monash Animal Research Platform and animals were recycled from a preceding gamma delta (γδ) T cell immunotherapy trial after confirmation γδ T cell frequencies had returned to baseline levels. Eight male macaques (*Macaca nemestrina*) (6–15 years old) were vaccinated with 100 μg of SARS-CoV-2 spike or RBD immunogens formulated with 200 μg of Monophosphoryl Lipid A (MPLA) liposomes (Polymun)^22^ intramuscularly in the right quadriceps. Twenty-eight days after priming, booster immunisations consisting of 100 μg S or RBD protein with 200 μg of MPLA and 1% tattoo ink were administered intramuscularly in both quadriceps. Although unlikely to be confounding, macaques were concurrently vaccinated in the right and left deltoids with HIV-trimeric envelope protein gp140 (SOSIP) immunogens (100 μg)^43,44^ formulated with MPLA and 1.0% tattoo ink (right deltoid only). Macaques were necropsied 14 days after booster vaccine administration. Twenty-four hours prior to necropsy, macaques received an intravenous infusion of autologous Vδ2^+^Vγ9^+^ T-cells labelled with CellTrace Blue (Life Technologies). Any CellTrace Blue^+^ cells were excluded from flow cytometric analysis of B or T cell populations.

### Flow cytometric detection of S and RBD-specific B cells

S protein was biotinylated using Bir-A (Avidity) and labelled by the sequential addition of streptavidin (SA) conjugated to PE (BD). RBD protein was directly labelled to APC using an APC Conjugation Lightning-Link Kit (Abcam). S and RBD probes were used for both murine and NHP studies. For murine studies, lymph nodes were mechanically homogenised into single-cell suspensions in RF10 media (RPMI 1640, 10% FCS, 1× penicillin–streptomycin–glutamine; Life Technologies). Isolated cells were stained with Aqua viability dye (Thermofisher) and Fc-blocked with a CD16/32 antibody (93; Biolegend; 2:75). Cells were then surface stained with S/RBD probes and the following antibodies: B220 BUV737 (RA3-6B2; BD; 1:300), IgD BUV395 (11–26 c.2a; BD; 1:300), CD45 APC-Cy7 (30-F11; BD; 1:300), SA BV786 (BD; 1:300), GL7 AF488 (GL7; Biolegend; 1:300), CD38 PE-Cy7 (90; Biolegend; 1:750), CD3 BV786 (145-2C11; Biolegend; 1:750) and F4/80 BV786 (BM; Biolegend; 1:150). Cells were washed twice with PBS containing 1% FCS and fixed with 1% formaldehyde (Polysciences).

For macaque studies, cryopreserved single cell suspensions were thawed, and stained with Aqua viability dye (Thermofisher). Cells were then surface stained with S/RBD probes and the following antibodies: IgD AF488 (polyclonal; Southern Biotech; 1:150), IgM BUV395 (G20-127; BD; 1:150), IgG BV786 (G18-145; BD; 1:75), CD14 BV510 (M5E2; Biolegend; 1:300), CD3 BV510 (OKT3; Biolegend; 1:600), CD8a BV510 (RPA-T8; Biolegend; 1:750), CD16 BV510 (3G8; Biolegend; 1:500), CD10 BV510 (HI10a; Biolegend; 1:750), CD20 APC-Cy7 (2H7; Biolegend; 1:150) (Biolegend) and SA BV510 (BD; 1:600). Cells were washed twice with PBS containing 1% FCS and fixed with 1% formaldehyde (Polysciences).

For intracellular transcription factor staining, cells were first stained with Aqua viability dye (Life Technologies), followed by S/RBD probes and surface antibodies: IgD AF488 (polyclonal; Southern Biotech; 1:150), IgG BV786 (G18-145; BD; 1:75), CD14 BV510 (M5E2; Biolegend; 1:300), CD3 BV510 (OKT3; Biolegend; 1:600), CD8a BV510 (RPA-T8; Biolegend; 1:750), CD16 BV510 (3G8; Biolegend; 1:500), CD10 BV510 (HI10a; Biolegend; 1/750), CD20 APC-Cy7 (2H7; Biolegend; 1:150) and SA BV510 (BD; 1:600). Cells were washed and permeabilised with Transcription Factor Buffer Set (BD) prior to BCL-6 PE-Cy7 (K112-91; BD; 2:25) and Ki-67 BUV395 (B56; BD; 3:50) staining. Cells were washed twice and resuspended in PBS containing 1% FCS. Samples were acquired on a BD LSR Fortessa using BD FACS Diva.

### Flow cytometric detection of ex vivo and antigen-specific TFH

For ex vivo TFH quantification from mice, freshly isolated LN single cell suspensions were stained with the following antibodies: Live/dead Red (Life Technologies), CD3 BV510 (145-2C11; Biolegend; 1:50), PD-1 BV786 (29 F.1A12; Biolegend; 1:100), CXCR5 BV421 (L138D7; Biolegend; 1:50), CD4 BUV737 (RM4-5; BD; 1:200), B220 BV605 (RA3-6B2; BD; 1:100) and F4/80 PE-Dazzle 594 (T45-2342; BD; 1:100). Cells were permeabilized with transcription factor staining buffer (BD Biosciences) and stained intracellularly with anti-BCL-6 Alexa647 (IG191E/A8; Biolegend; 1:10). Non-human primate LN suspensions and PBMC were stained with the same protocol, using the following antibodies: Live/dead Aqua (Life Technologies), CD3 Alexa700 (SP34-2; BD; 1:100), PD-1 BV421 (EH12.2H7; Biolegend; 1:50), CXCR5 PE (MU5UBEE; ThermoFisher; 1:50), CD4 BV605 (L200; BD; 1:100), CD20 BV510 (2H7; BD; 1:100), CD8 BV650 (RPA-T8; Biolegend; 1:400), CD95 BUV737 (DX2; BD; 1:200), ICOS PerCP-Cy5.5 (C398.4A; Biolegend; 1:50), CD69 FITC (FN50; Biolegend; 1:200), CCR6 BV785 (G034E3; Biolegend; 1:100), CXCR3 Pe-Dazzle594 (G02H57; Biolegend; 1:50), BCL-6 APC (IG191E/A8; Biolegend; 1:10) and Ki67 BUV395 (B56; BD; 1:33).

To identify antigen-specific TFH cells, LN cell or PBMC suspensions were cultured in RF10 media for 18 h at 37 °C. Cryopreserved NHP samples were rested for 4 h at 37 °C prior to stimulation, while murine samples were processed fresh. Samples were stimulated with a peptide pool (15mers overlapping by 11, 2 μg/peptide/mL) comprising the SARS-CoV-2 RBD or SARS-CoV-2 S (without the RBD), or a DMSO control. In some experiments, cells were stimulated with individual peptides at 2 μg/mL: P7; PPAYTNSFTRGVYYP; P16, NVTWFHAIHVSGTNG; P99, TNVYADSFVIRGDEV; P121, NGVEGFNCYFPLQSY; P129, LSFELLHAPATVCGP; P277, TQRNFYEPQIITTDN; P280, TDNTFVSGNCDVVIG. At the time of stimulation, an anti-mouse CD154 PE mAb (MR1; Biolegend; 1:500) or anti-human CD154 APC-Cy7 (TRAP1; BD; 1:300) was added to all culture conditions. After stimulation, cells were washed twice in PBS and stained with viability dye (Red or Aqua, Life Technologies) according to the manufacturer’s instructions. Mouse cells were then stained with CD3 BV510 (145-2C11; Biolegend; 1:50), CD25 BB515 (PD61; BD; 1:50), PD-1 BV786 (29F.1A12; Biolegend; 1:100), CXCR5 BV421 (L138D7; Biolegend; 1:50), CD4 BUV737 (RM4-5; BD; 1:200), OX-40 PeCy7 (OX-86; Biolegend; 1:100), B220 BV605 (RA3-6B2; BD; 1:100) and F4/80 PE-Dazzle 594 (T45-2342; BD; 1:100) before being washed and fixed. NHP cells were stained with the following antibodies: CD3 Alexa700 (SP34-2; BD; 1:100), PD-1 BV421 (EH12.2H7; Biolegend; 1:50), CXCR5 PE (MU5UBEE; ThermoFisher; 1:50), CD4 BV605 (L200; BD; 1:100), CD20 BV510 (2H7; BD; 1:100), CD8 BV650 (RPA-T8; Biolegend; 1:400), CD95 BUV737 (DX2; BD; 1:200), CD28 BV711 (CD28.2; BD; 1:25), CCR6 BV785 (G034E3; Biolegend; 1:100), CXCR3 Pe-Dazzle594 (G02H57; Biolegend; 1:50), CD25 APC (BC96; Biolegend; 1:50) and OX-40 BUV395 (L106; BD; 1:33). Samples were acquired on a BD LSR Fortessa using BD FACS Diva.

### Quantification of peptide-induced cytokine secretion

Macaque PBMC were stimulated for 18 h with DMSO (negative control), SEB (positive control) or peptide pools spanning the RBD or non-RBD epitopes of the S antigen. Supernatants were collected and frozen at –80 °C. Cytokine concentrations were determined using the Legendplex Non-human Primate 10-plex Th Cytokine Panel according to manufacturer’s instructions. All samples were run in duplicate. Sample analyte concentrations were determined by standard curve interpolation using Legendplex data analysis software v8.0. After background subtraction, data from RBD- and non-RBD-specific wells were added for each animal to generate the cumulative cytokine response to peptides spanning the entire S antigen.

### B-cell receptor sequencing and analysis

B cell receptor sequences were recovered from GC B cells (B220 + IgD-GL7 +) in the draining iliac lymph node of C57BL/6 mice (*n* = 3) 14 days after a single immunisation with S. Single cells were sorted using a BD Aria II into 96-well plates and subject to cDNA generation and multiplex PCR and sanger sequencing^45^. Briefly, plates containing single sorted cells were thawed and cDNA prepared using Superscript III reverse transcriptase (Invitrogen) with incubation conditions of 42 °C, 5 min; 25 °C, 5 min; 50 °C, 60 min; 94 °C, 5 min; 4 °C. Primary and secondary PCR reactions for recovery of murine heavy chain immunoglobulins was performed using HotStart Taq (Qiagen) and multiplex PCR primers (Supplementary Table 1A). Cycling conditions for primary PCRs were one cycle of 94 °C, 5 min; 50 cycles of 94 °C, 30 s; 46 °C, 20 s; 72 °C, 55 s; one cycle of 72 °C, 10 min; 4 °C. Cycling conditions for secondary PCRs were one cycle of 94 °C, 5 min; 50 cycles of 94 °C, 30 s; 57 °C, 20 s; 72 °C, 55 s; one cycle of 72 °C, 10 min; 4 °C. Secondary PCR products were sequenced using the secondary reverse constant chain primer using standard sanger sequencing (Macrogen).

For macaques, a single-cell suspension was prepared from the draining iliac lymph node of a single animal 14 days after a second immunisation with S. Single S- (S^+^RBD^−^) and RBD-specific (S^+^RBD^+^) IgG^+^ B cells were stained as above and sorted using a BD Aria II into 96-well plates. Recombined immunoglobulin heavy chain genes were subject to cDNA generation and multiplex PCR and sanger sequencing^46^. Briefly, cDNA from single sorted cells was prepared using Superscript III (Invitrogen) with incubation conditions of 42 °C, 5 min; 25 °C, 5 min; 50 °C, 60 min; 94 °C, 5 min; 4 °C. Primary and secondary PCRs were performed using HotStart Taq (Qiagen) and a set of nested multiplex PCR primers (Supplementary Table 1A). Cycling conditions for primary PCRs were one cycle of 95 °C, 5 min; 49 cycles of 94 °C, 30 s; 50 °C, 45 s; 72 °C, 45 s; one cycle of 72 °C, 10 min; 4 °C. Cycling conditions for secondary PCRs were one cycle of 94 °C, 5 min; 35 cycles of 94 °C, 30 s; 60 °C, 45 s; 72 °C, 45 s; one cycle of 72 °C, 10 min; 4 °C. Secondary PCR products were sequenced using standard sanger sequencing (Macrogen). Productive, recombined heavy (V-D-J) and light chain (V-J) immunoglobulin sequences were analysed using IMGT V-quest^47^.

### ELISA

Antibody binding to coronavirus S or RBD proteins was tested by ELISA. 96-well Maxisorp plates (Thermo Fisher) were coated overnight at 4 °C with 2 μg/mL recombinant S, RBD or OVA proteins. After blocking with 1% FCS in PBS, duplicate wells of serially diluted plasma were added and incubated for 2 h at room temperature. Plates were washed prior to incubation with HRP-conjugated secondary antibodies for the mouse (1:10000; anti-mouse IgG; KPL), macaque (1:10000; anti-macaque IgG; Kerafast) or human (1:20000; anti-human IgG; Agilent) for 1 h at room temperature. Plates were washed and developed using TMB substrate (Sigma) and read at 450 nm. Endpoint titres were calculated as the reciprocal serum dilution giving signal 2✕ background using a fitted curve (four parameter log regression).

### Blocking ELISA

Plates were coated as before with 2 μg/mL recombinant S and blocked with 1% FCS in PBS. Serially diluted sera (mouse) or plasma (non-human primate, convalescent COVID-19 donors) was pre-incubated with 10 mg/ml of recombinant BSA, S, RBD or NTD proteins in 1% FCS/PBS for 2 h, before addition to S-coated plates for 15 min. Plates were washed prior to incubation with HRP-conjugated secondary antibodies for the mouse (1:10000; anti-mouse IgG; KPL/SeraCare), macaque (1:10000; anti-macaque IgG; Kerafast) or human (1:20000; anti-human IgG; Agilent) and developed and read as above.

### ACE2-RBD inhibition ELISA

An ELISA to measure the ability of plasma antibodies to block the interaction between recombinant human ACE2 and SARS-CoV-2 RBD was performed^17^. The human ACE2 (residues 19–613) ectodomain with C-terminal His-tag (kindly provided by Merlin Thomas) was expressed in Expi293 cells and purified using Ni-NTA and size-exclusion chromatography. Briefly, 96-well Maxisorp plates (Thermo Fisher) were coated overnight at 4 °C with 2.5 µg/ml of recombinant RBD protein in carbonate–bicarbonate coating buffer (Sigma). After blocking with PBS containing 1% BSA, duplicate wells of serially diluted plasma (1:25 to 1:102,400) were added and incubated for 1 h at room temperature. Plates were then incubated with 1 µg/ml of biotinylated recombinant ACE2 protein for 1 h at room temperature followed by incubation with HRP-conjugated streptavidin (Thermo Fisher Scientific) for 1 h at room temperature. Plates were developed with TMB substrate (Sigma), stopped with 0.15 M sulphuric acid and read at 450 nm. %Inhibition was plotted against plasma dilutions and the area under the curve (AUC) was calculated using Graphpad Prism.

### Microneutralisation assay

SARS-CoV-2 isolate CoV/Australia/VIC01/2020^48^ was passaged in Vero cells and stored at −80 °C. Plasma was heat inactivated at 56 °C for 30 min. Plasma was serially diluted 1:20 to 1:10,240 before the addition of 100 TCID_50_ of SARS-CoV-2 in MEM/0.5% BSA and incubation at room temperature for 1 h. Residual virus infectivity in the plasma/virus mixtures was assessed in quadruplicate wells of Vero cells incubated in serum-free media containing 1 μg/ml of TPCK trypsin at 37 °C and 5% CO_2_; viral cytopathic effect was read on day 5. The neutralising antibody titre was calculated using the Reed–Muench method^49,50^.

### Statistics

Data is generally presented as median +/− interquartile range or range. Statistical significance was assessed by Mann–Whitney U tests. Curve fitting was performed using four parameter logistic regression. All statistical analyses were performed using Prism (GraphPad). Flow cytometry data was analysed in FlowJo v9 or v10.

### Reporting summary

Further information on research design is available in the Nature Research Reporting Summary linked to this article.

## Supplementary information

Supplementary Information

Reporting Summary

## Data Availability

The data that support the findings of this study are available in the Source Data File provided with this paper, and are otherwise available from the corresponding author upon reasonable request. Source data are provided with this paper.

## Source data

Source Data
