# Supplementary material for: Immunogenicity of prime-boost protein subunit vaccine strategies against SARS-CoV-2 in mice and macaques
Source: Nat Commun. 2021 Mar 3;12:1403. doi: 10.1038/s41467-021-21665-8 (PMC7930087; doi:10.1038/s41467-021-21665-8)
Supplement: Supplementary file 1 — Supplementary Information [file 41467_2021_21665_MOESM1_ESM.pdf]

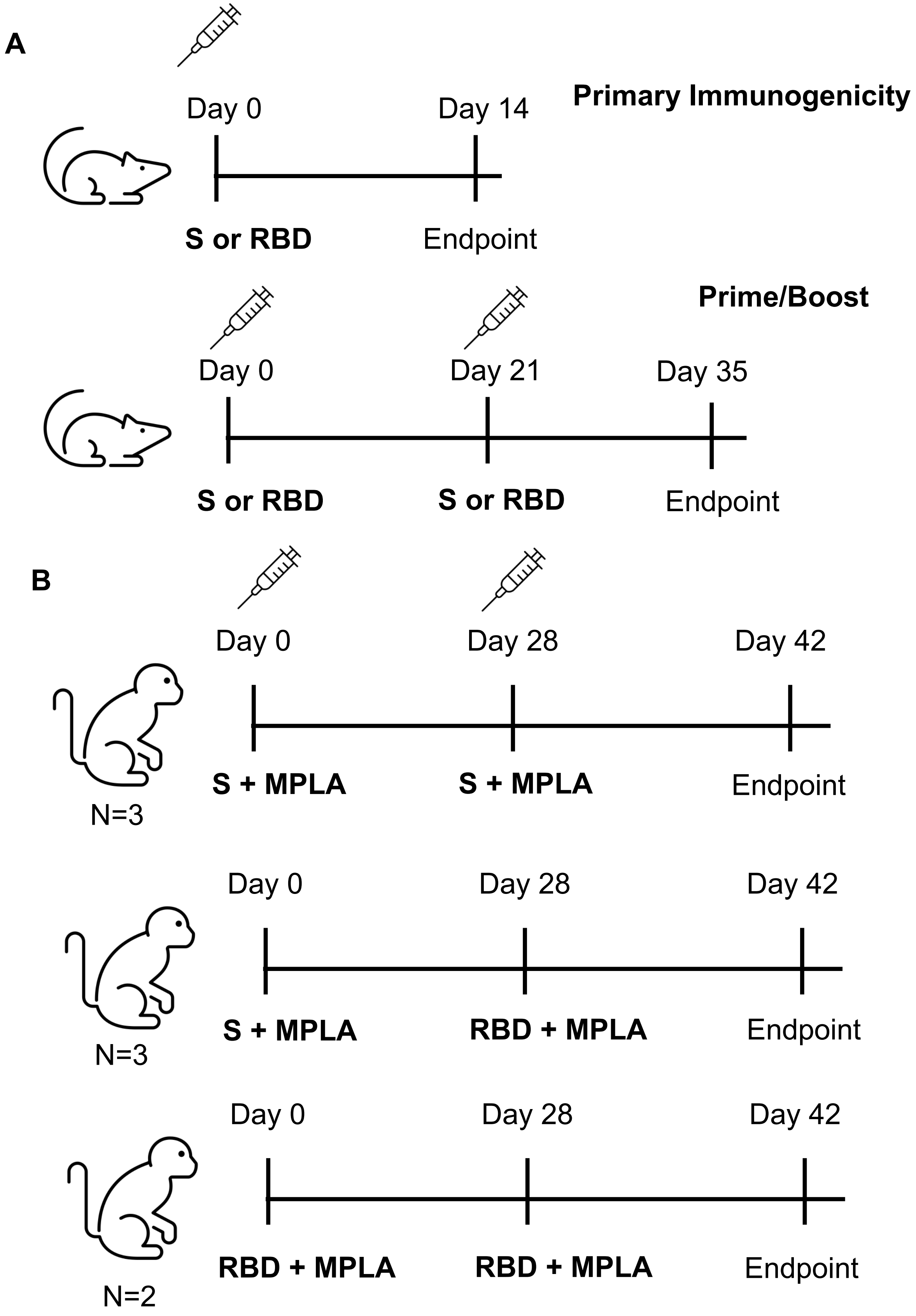

Supplemental Figure 1. Animal trial vaccination schedule. (A) Mice were immunised with recombinant S or RBD proteins for assessment of either primary immunogenicity or a prime/boost regimen. (B) Three groups of pigtail macaques were vaccinated with combinations of S or RBD antigens in a prime/boost regimen, with peripheral blood and lymph node samples collected at day 42.

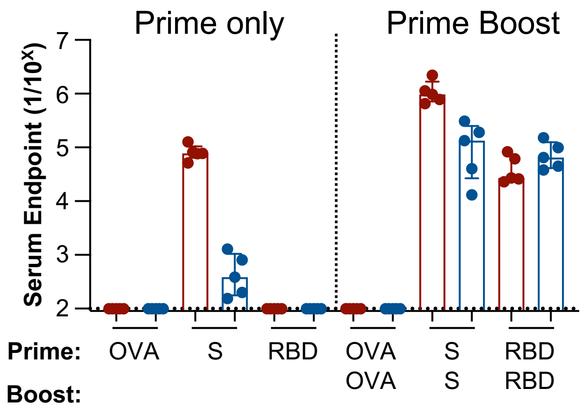

Supplementary Figure 2. Comparison of serological responses to S and RBD immunogens at day 28. Mice were serially immunised intramuscularly at a 21-day interval with S, RBD or OVA proteins and immune responses assessed at 28 days post-prime or post-boost (N=5 per group). Reciprocal serum endpoint dilutions of S- (red) or RBD-specific (blue) IgG were measured by ELISA. Error bars indicate interquartile range.

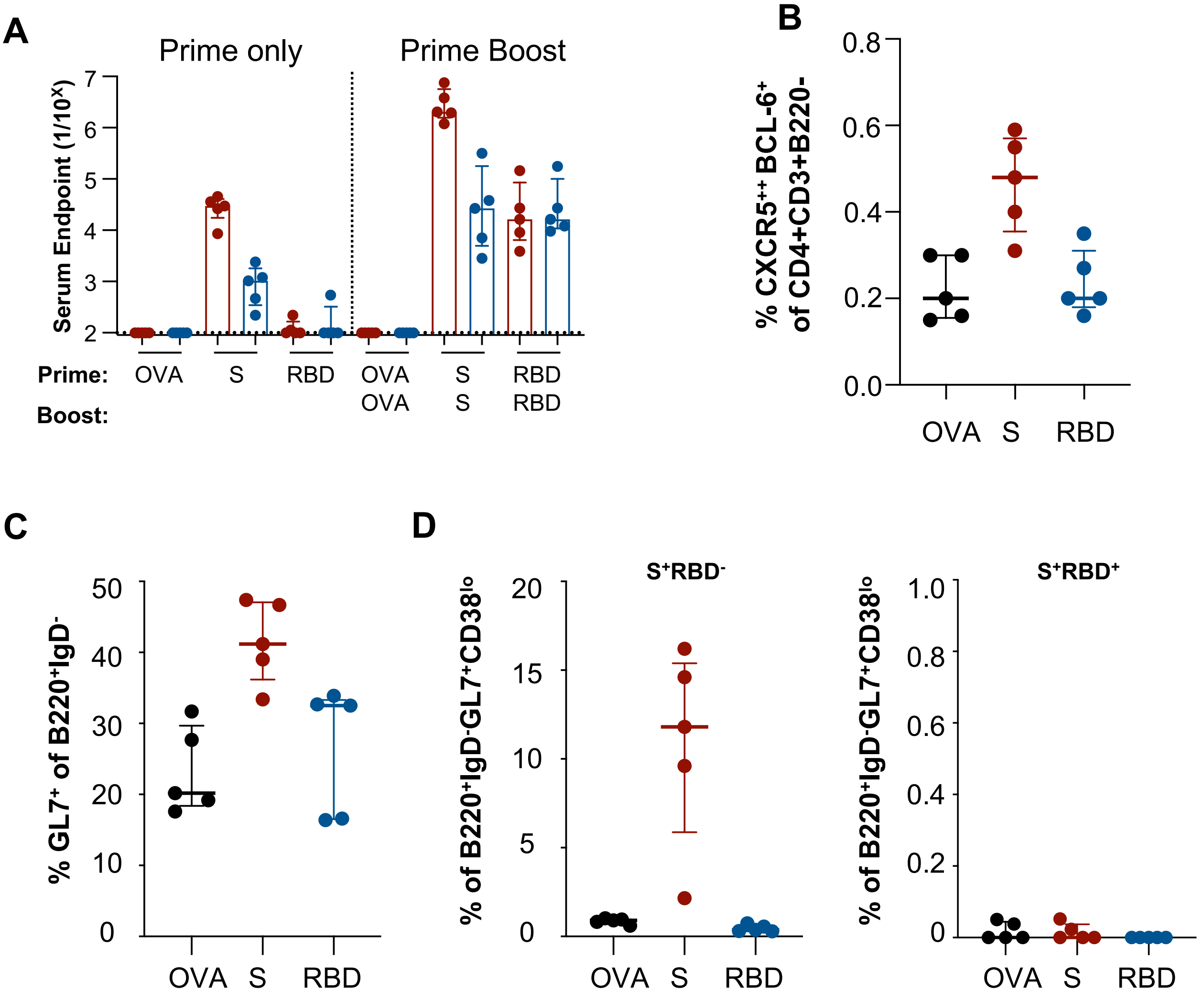

Supplemental Figure 3. Primary immunogenicity of SARS-CoV-2 subunit proteins formulated with MPLA. Mice were immunised intramuscularly at a 21-day interval with S (red), RBD (blue) or OVA (black) immunogens formulated with MPLA adjuvant and immune responses assessed 14 days post-primary and/or secondary immunisation (n = 5). **(A)** Reciprocal serum endpoint dilutions of S- (red) or RBD-specific (blue) were measured by ELISA. Dotted lines denote the detection cut off (1:100 dilution). Mice immunised once (prime only) were assessed for **(B)** frequency of TFH cells ($CXCR5^{++}BCL\text{-}6^{+}CD4^{+}CD3^{+}B220^{-}$), **(C)** germinal centre activity in draining lymph node by GL7 expression in $B220^{+}IgD^{-}$ B cells and **(D)** frequency of germinal centre B cells ($B220^{+}IgD^{-}GL7^{+}CD38^{lo}$) specific for Spike ($S^{+}RBD^{-}$) or RBD ($S^{+}RBD^{+}$) probes. Data is presented as median ± IQR.

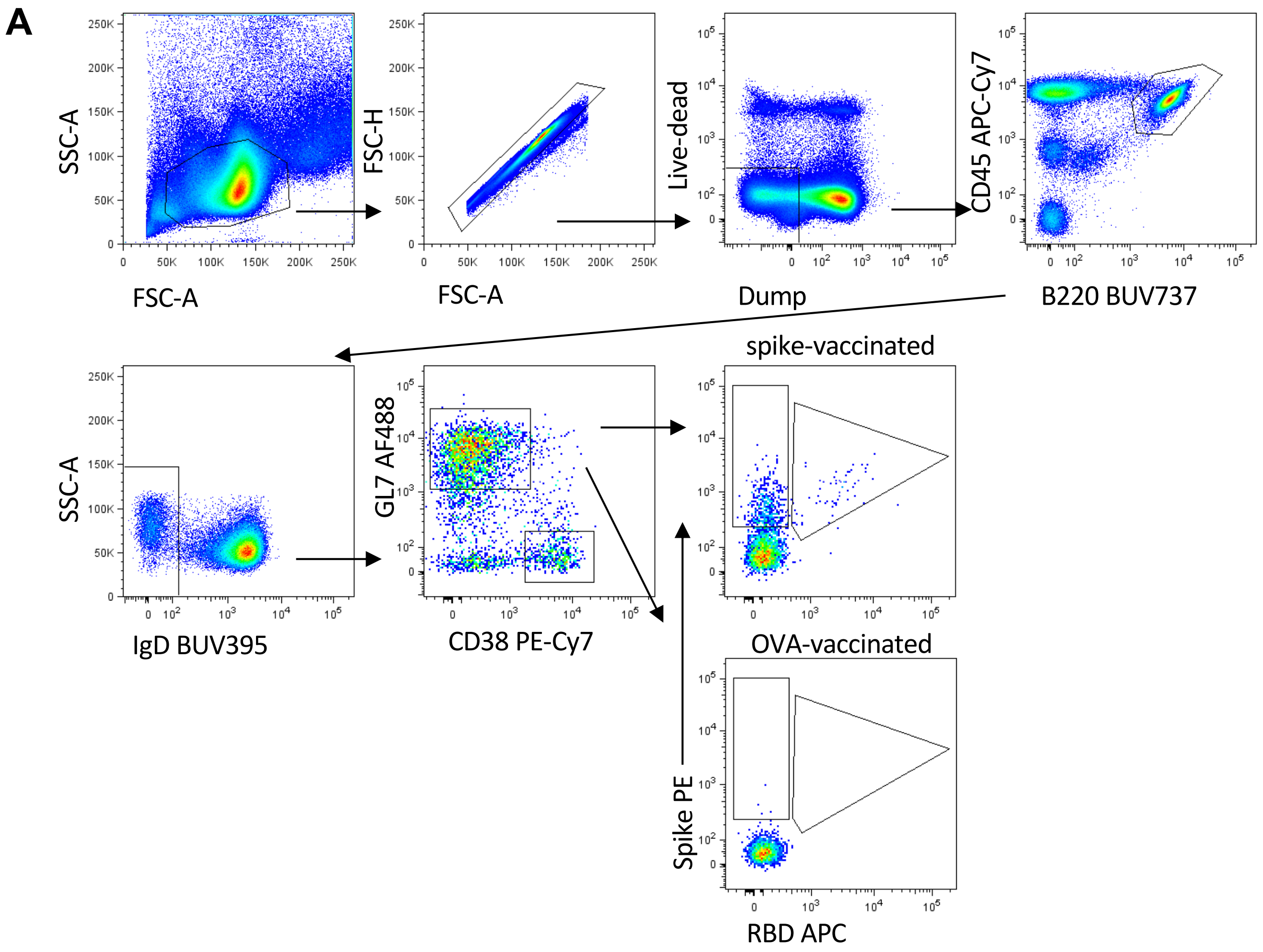

Supplemental Figure 4. Identification of bulk and antigen-specific mouse germinal centre B cells. Lymphocytes were identified by FSC-A vs SSC-A gating, followed by doublet exclusion (FSC-A vs FSC-H). Live and CD3-F4/80-streptavidin- (dump channel) cells were gated and $CD45^+B220^+IgD^-$ B cells identified. Germinal centre ($GL7^+CD38^{lo}$) B cells were then assessed for binding to SARS-CoV-2 spike (S) and/or SARS-CoV-2 RBD probes. Gating strategy corresponds to data presented in Figure 1C-D and Figure 2D-E.

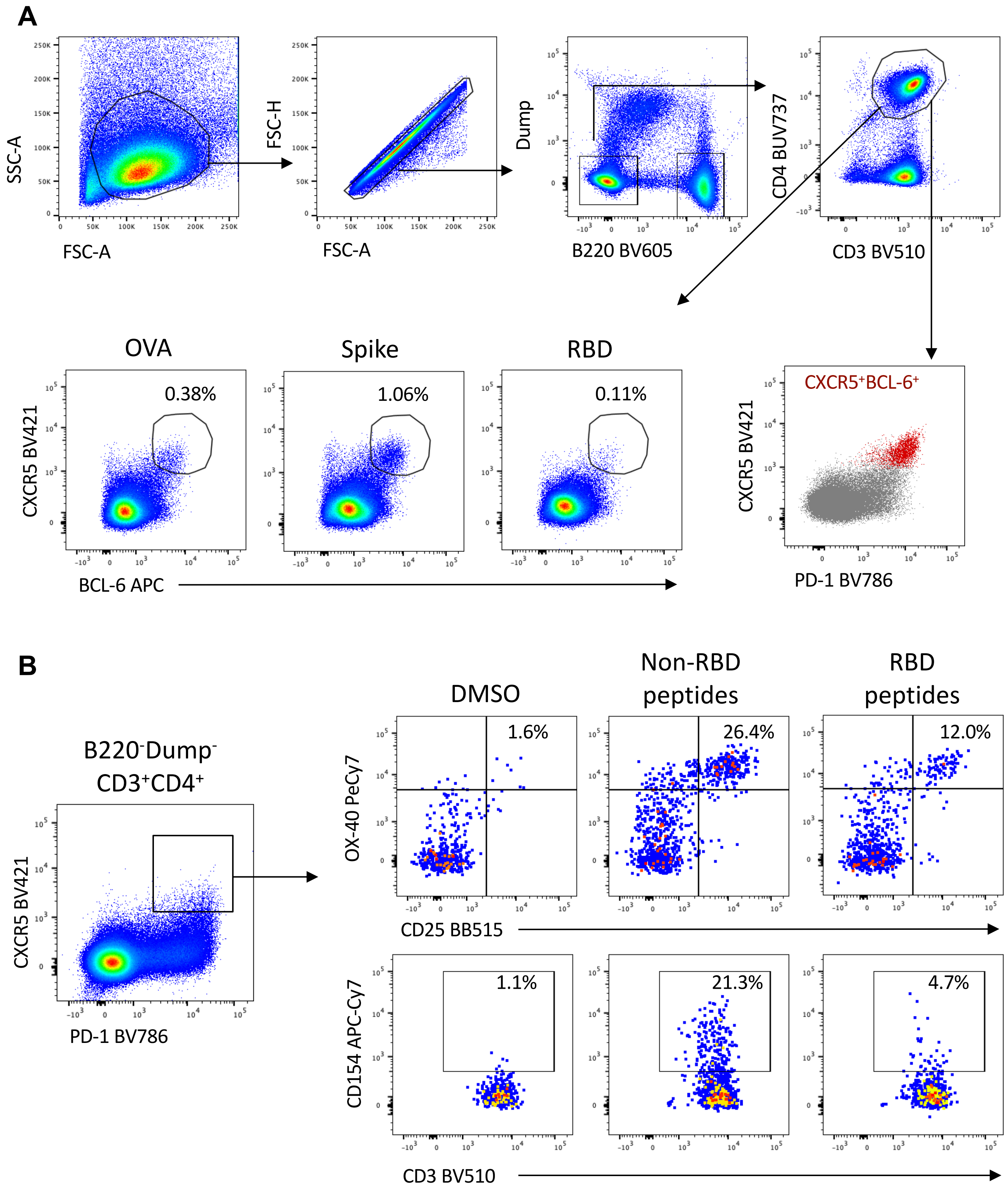

Supplemental Figure 5. Identification of bulk and antigen-specific mouse TFH cells. **(A)** Gating strategy for ex vivo identification of lymph node TFH cells, based on BCL-6 and CXCR5 expression. Comparison of bulk $CD4^+$ (grey) and TFH cells (red) confirms the PD-1^{hi} phenotype of the BCL-6^+CXCR5^{hi} population. Gating corresponds to data presented in Figure 1E and 2F. **(B)** Identification of S-specific TFH (OX-40^{++}CD25^+ or CD154^+) following peptide pool stimulation of lymph node suspensions from S-vaccinated animals (plots represent pooled lymph nodes from 3 animals). Gating corresponds to data presented in Figure 1F.

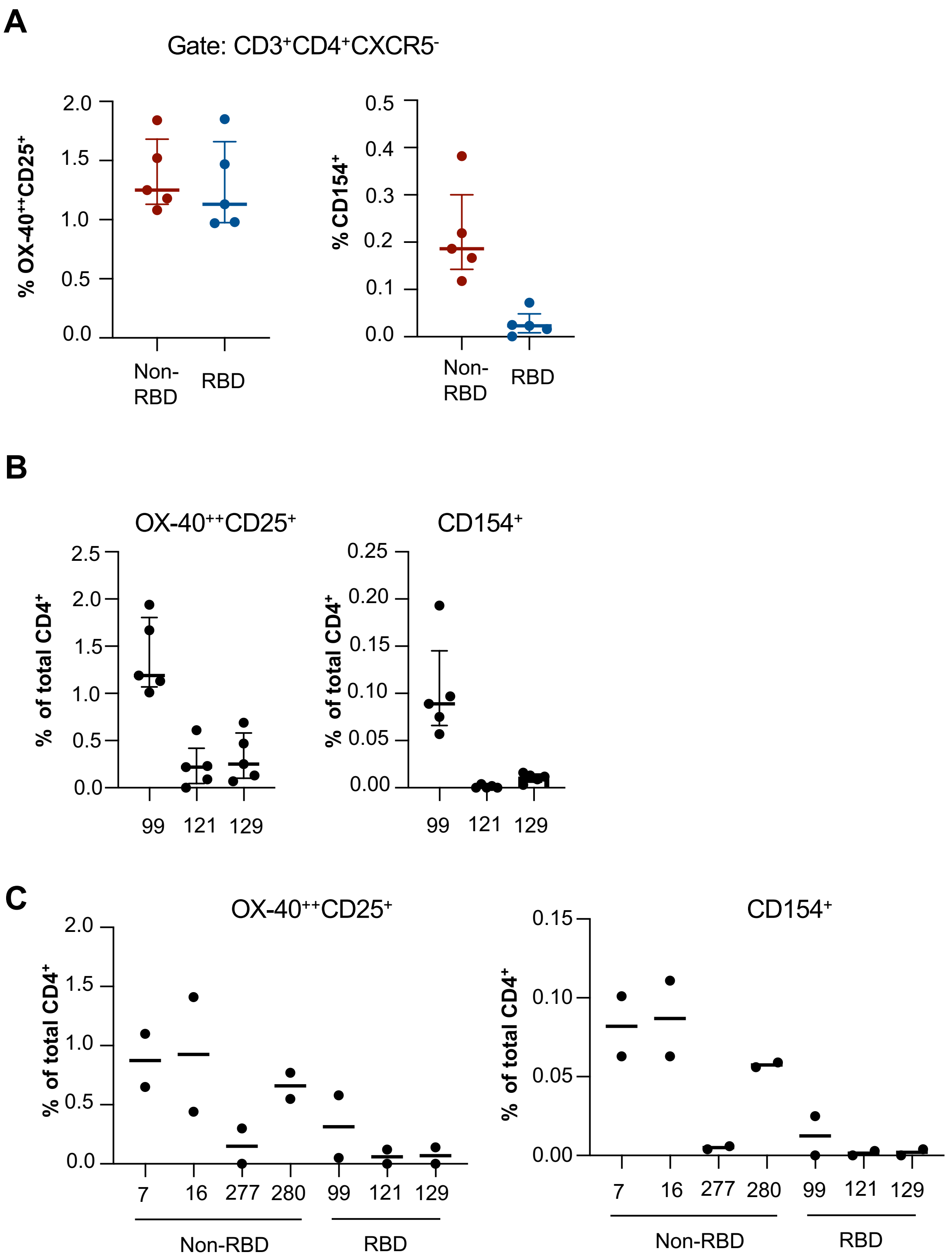

Supplemental Figure 6. SARS-CoV-2 RBD immunogenic T cell epitopes. (A) Quantification of OX-40++CD25+ or CD154+ CD4 T cells with specificity for non-RBD (red) or RBD-derived (blue) epitopes following primary vaccination with S protein (n=5 mice). **(B)** Screening of RBD-derived 15-mer peptides identified 3 T cell epitopes recognized by C57BL/6 mice. Graphs indicate the frequency of OX-40^{++}CD25^{+} or CD154^{+} CD4^{+} cells following in vitro peptide re-stimulation of lymph node suspensions from RBD-vaccinated mice (n=5 individual mice). **(C)** Screening of non-RBD peptides identified multiple T cell epitopes immunogenic in C57BL/6 mice, including peptides 7, 16, 277 and 280. Graphs indicate the frequency of peptide-specific CD4^{+} T cells in pooled lymph node suspensions of S-vaccinated mice (each data point represents a pool of 5 mice). Data is presented as median ± IQR.

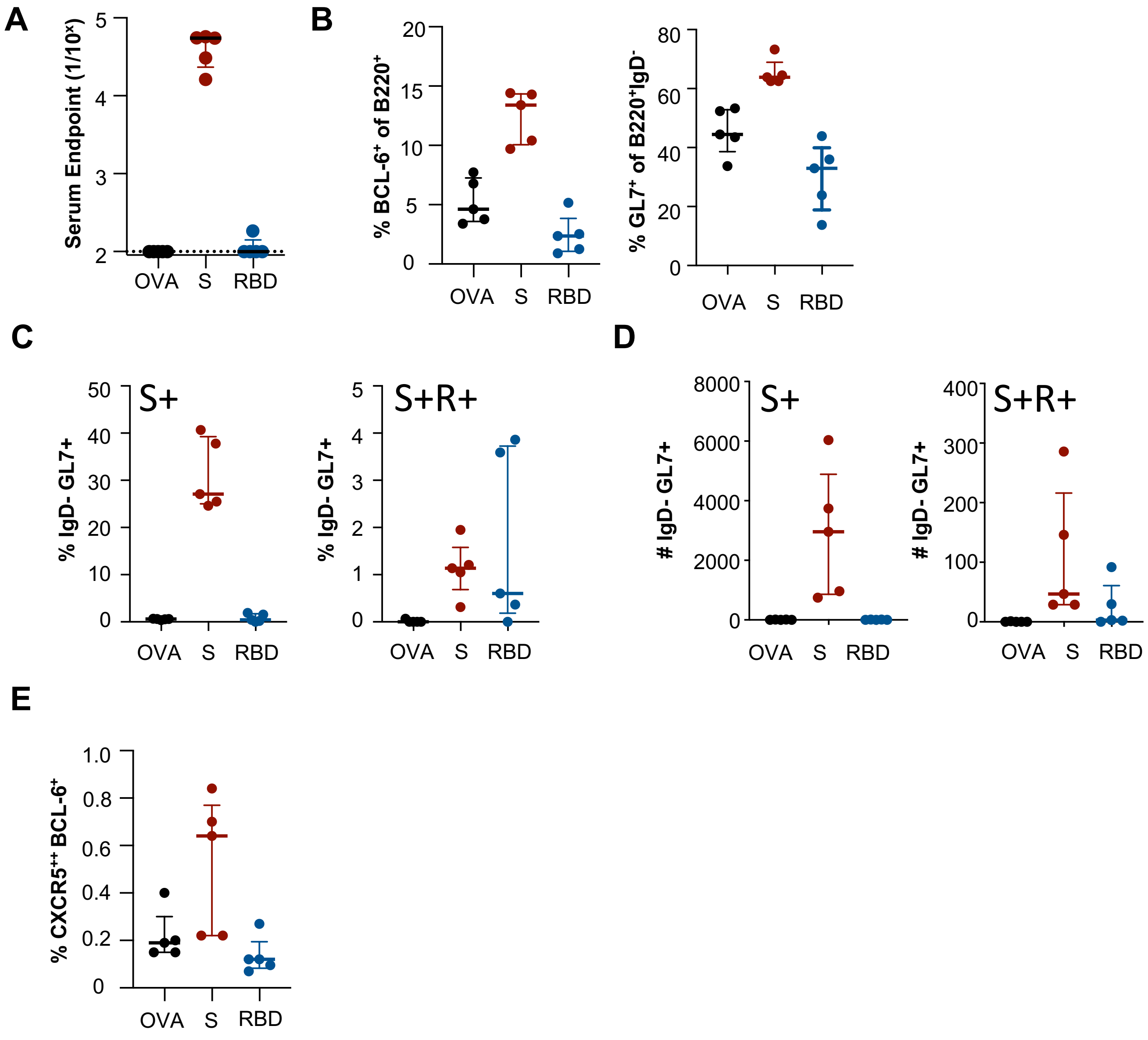

Supplemental Figure 7. Primary immunogenicity of SARS-CoV-2 subunit proteins in BALB/c mice. Mice were immunised intramuscularly with S, RBD or OVA immunogens and immune responses assessed 14 days post-immunisation (n = 5). **(A)** Reciprocal serum endpoint dilutions of S- (red), RBD- (blue) or OVA-specific IgG (black) were measured by ELISA. Dotted lines denote the detection cut off (1:100 dilution). **(B)** Draining lymph node germinal centre activity assessed by BCL-6 expression in $B220^+$ B cells or GL7 expression in $B220^+IgD^-$ B cells. **(C)** Frequency and **(D)** absolute counts of germinal centre B cells ($B220^+IgD^-GL7^+CD38^{lo}$) specific for spike ($S^+RBD^-$) or RBD ($S^+RBD^+$) probes. **(E)** Frequency of TFH cells ($CXCR5^{++}BCL\text{-}6^+CD4^+CD3^+B220^-$). Data is presented as median ± IQR.

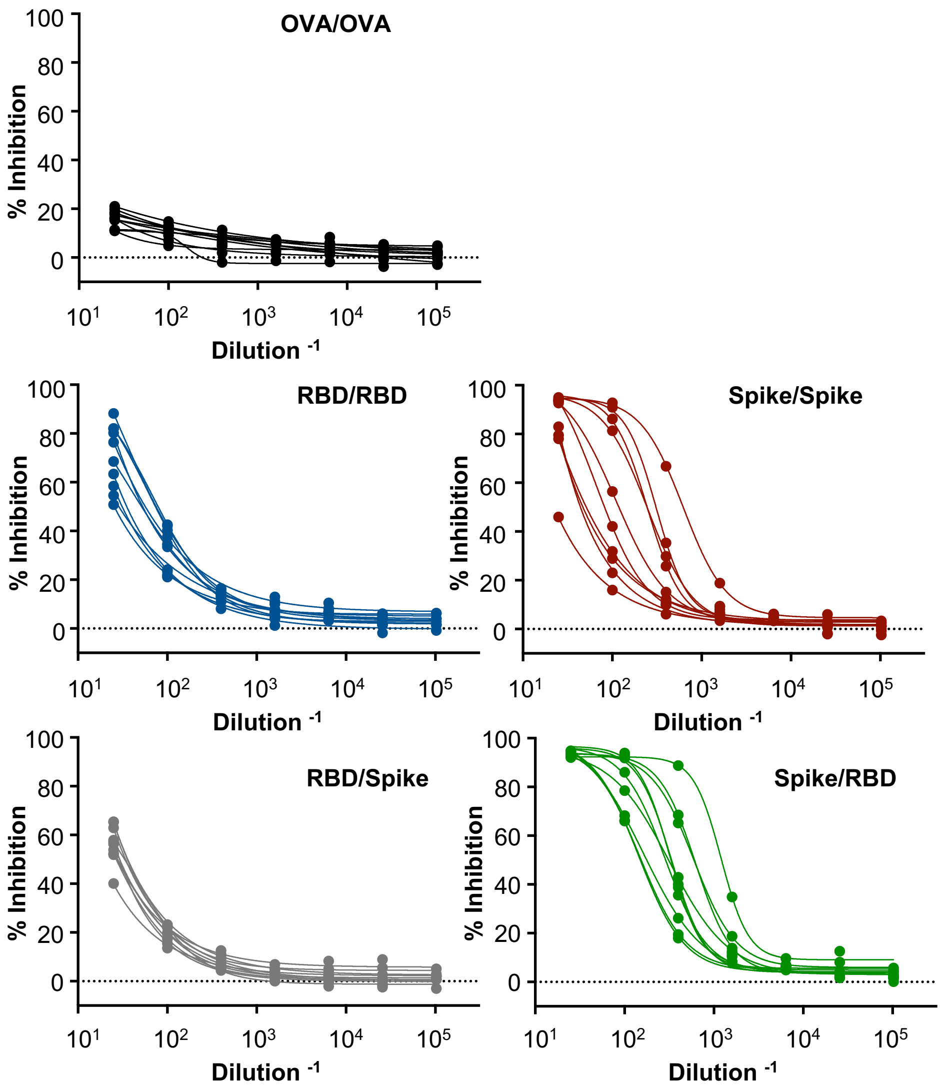

Supplemental Figure 8. Inhibition of ACE2-RBD engagement by serum antibodies in immunised C57BL/6 mice. Mice were serially immunised intramuscularly at a 21-day interval with S, RBD or OVA proteins and immune responses assessed 14 days post-boost**.** The capacity for serum antibodies from immunised mice (n = 10 per group) to inhibit the interaction of recombinant SARS-CoV-2 RBD and human ACE2 was assessed by ELISA.

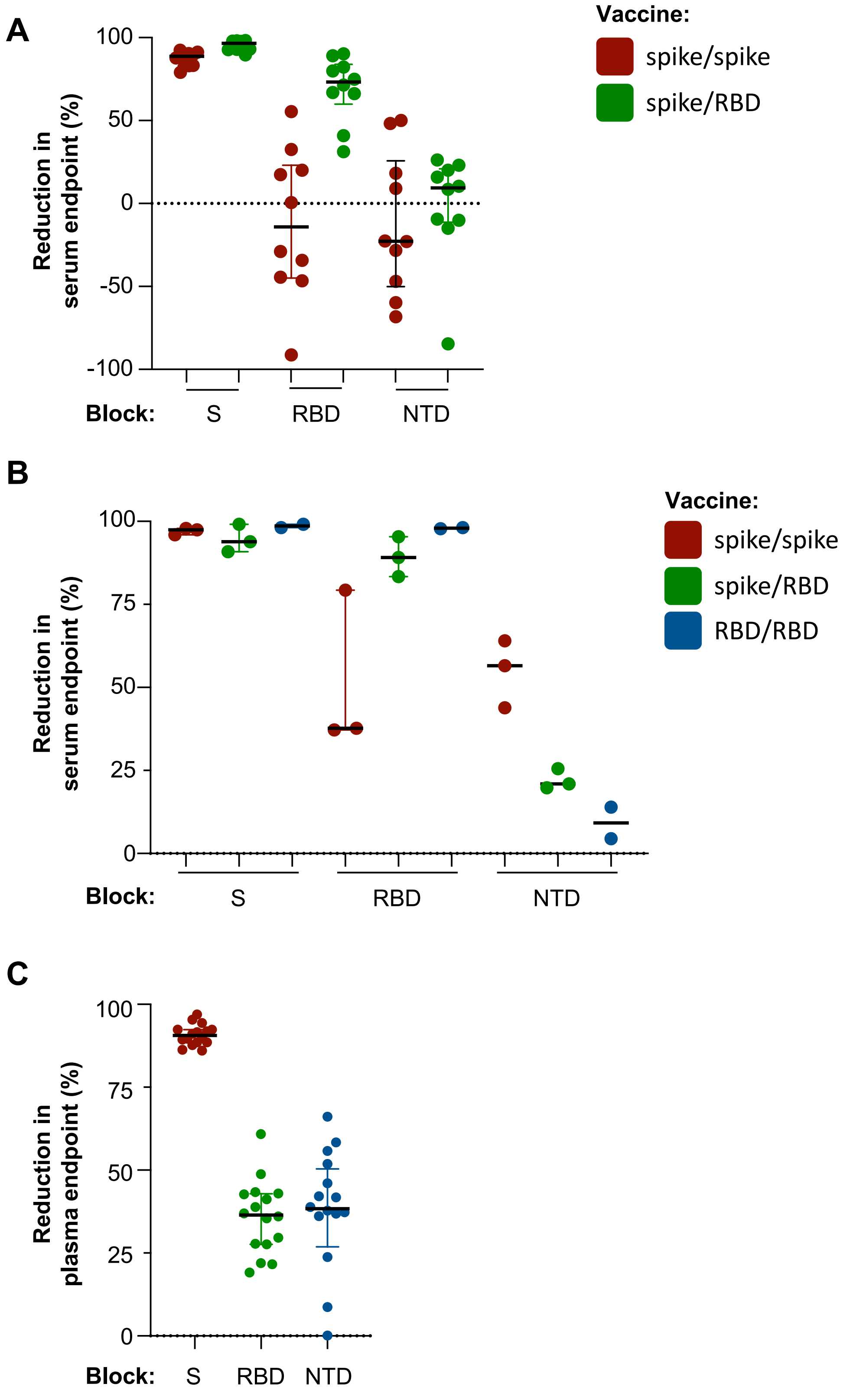

Supplemental Figure 9. Indicative specificity mapping of S-specific antibody responses
The relative proportion of RBD or NTD reactivity in the S-specific antibody response in immunised (**A**) mice (N=10) and (**B**) non-human primates (N=3 spike/spike or spike/RBD; N=2 RBD/RBD), or (**C**) a random selection of convalescent COVID-19 subjects (N=16), was assessed using a blocking ELISA format. Percentage reductions in serum or plasma endpoint dilutions were calculated after blocking with S, RBD or NTD proteins and calculated relative to a BSA blocked control.

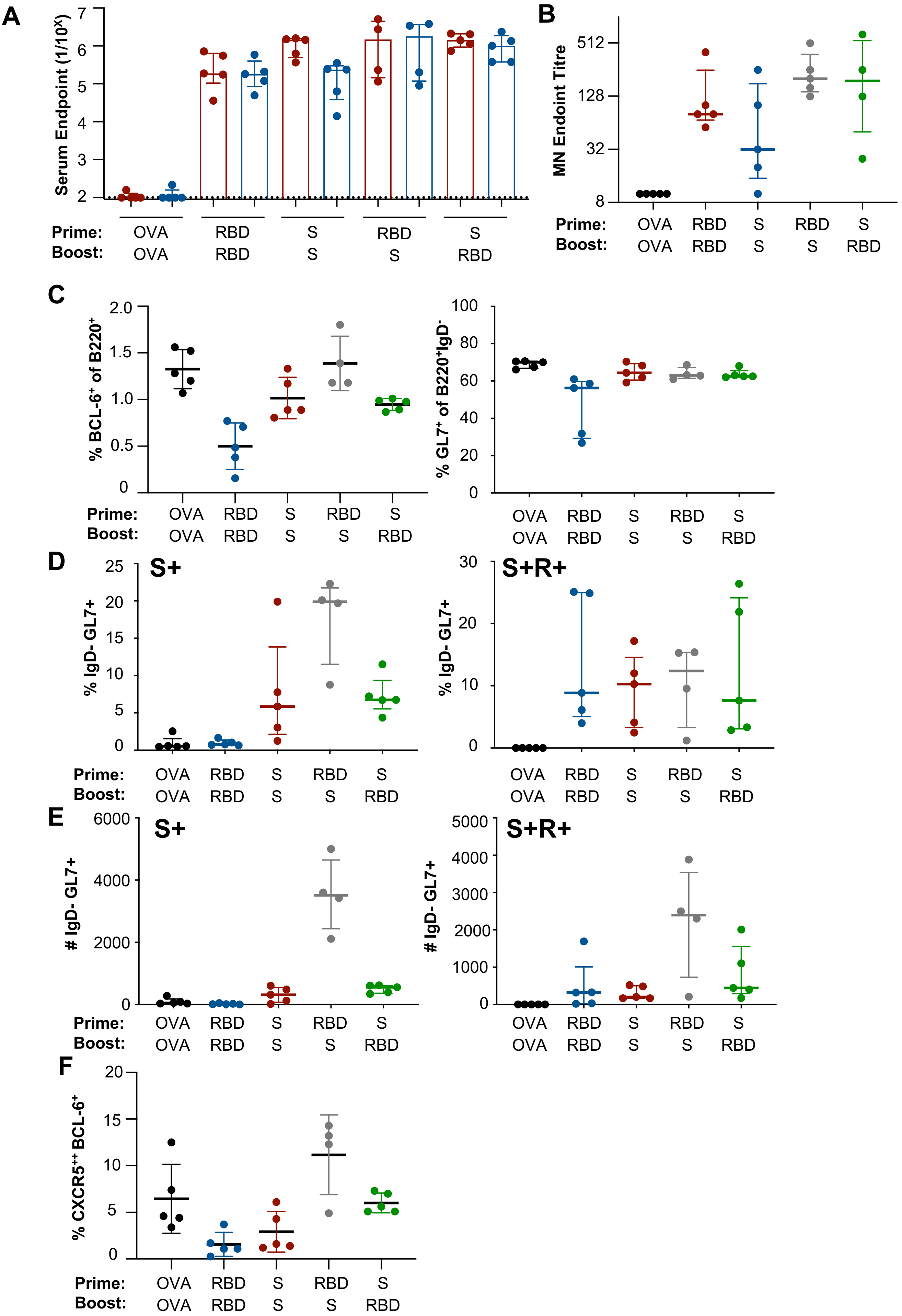

A
Serum Endpoint (1/10^X)
7
6
5
4
3
2
Prime:
Boost:
OVA
OVA
RBD
RBD
S
S
RBD
S
S
RBD
B
MN Endoint Titre
512
128
32
8
C
% BCL-6+ of B220+
2.0
1.5
1.0
0.5
0
% GL7+ of B220+IgD-
100
80
60
40
20
D
S+
S+R+
% IgD- GL7+
25
20
15
10
30
E
IgD- GL7+
6000
4000
2000
5000
3000
1000
F
% CXCR5++ BCL-6+

Supplemental Figure 10. Prime-boost immunisation of SARS-CoV-2 subunit proteins in BALB/c mice. Mice were serially immunised intramuscularly at a 21-day interval with S, RBD or OVA proteins (S/S red, RBD/RBD blue, OVA/OVA black, RBD/S grey, S/RBD green) and immune responses assessed 14 days post-boost (n = 5, except group RBD-spike n = 4). **(A)** Reciprocal serum endpoint dilutions of S- (red) or RBD-specific (blue) were measured by ELISA. Dotted lines denote the detection cut off (1:100 dilution). **(B)** Neutralisation activity in the serum was assessed using a microneutralisation assay. **(C)** Draining lymph node germinal centre activity assessed by BCL-6 expression in $B220^+$ B cells or GL7 expression in $B220^+IgD^-$ B cells. **(D)** Frequency and **(E)** absolute counts of of germinal centre B cells ($B220^+IgD^-GL7^+CD38^{lo}$) specific for spike ($S^+RBD^-$) or RBD ($S^+RBD^+$) probes. **(F)** Frequency of TFH cells ($CXCR5^{++}BCL\text{-}6^+CD4^+CD3^+B220^-$). Data is presented as median ± IQR.

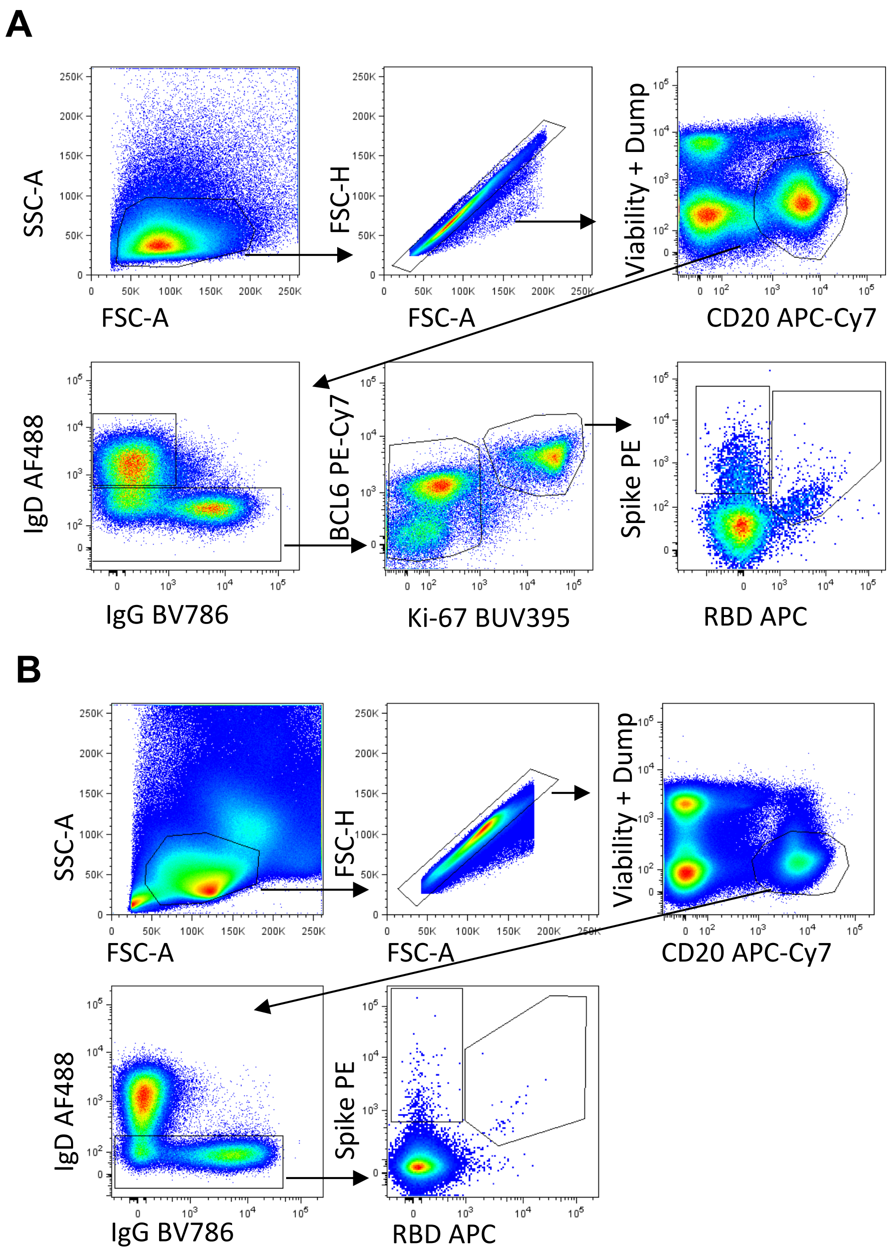

Supplemental Figure 11. Macaque B cell gating strategy. Gating of antigen-specific **(A)** germinal centre B cells in lymph nodes or **(B)** circulating memory B cells in PBMC samples. Lymphocytes were identified by FSC-A vs SSC-A gating, followed by doublet exclusion (FSC-A vs FSC-H), and gating on dump$^-$ (CD3$^-$CD8$^-$CD14$^-$CD10$^-$CD16$^-$ streptavidin$^-$) live CD20$^+$ B cells. **(A)** Antigen-specific germinal center B cells were identified from class-switched IgD- B cells and intracellular expression of BCL6 and Ki-67. Alternatively, **(B)** circulating memory B cells in PBMC samples were identified as CD20$^+$IgD$^-$. Antigen specificity was determined by binding to SARS-CoV-2 spike (S) and/or SARS-CoV-2 RBD probes. Gating corresponds to data presented in Figure 3D-E.

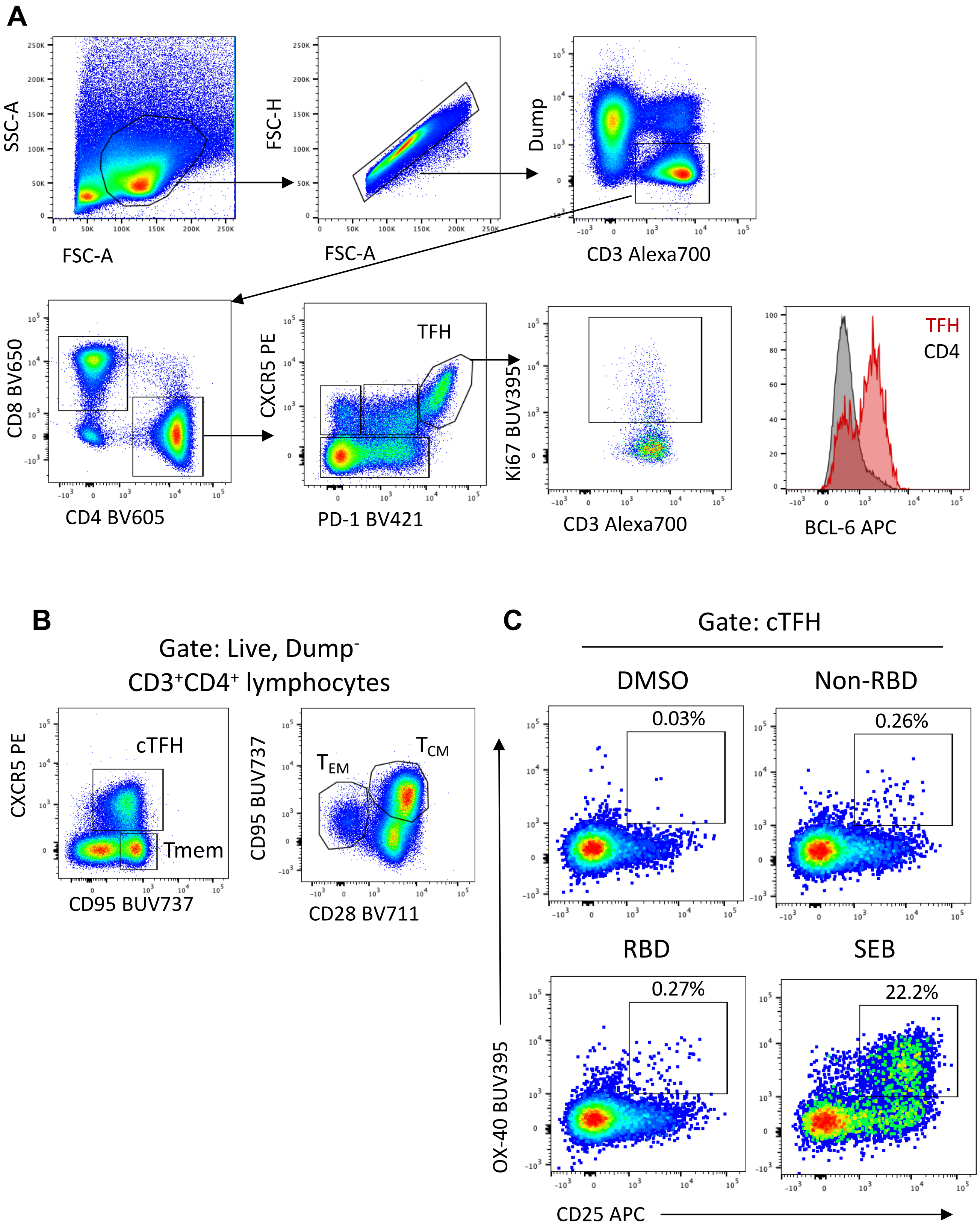

Supplemental Figure 12. Macaque T cell gating strategy. **(A)** Gating of lymph node GC TFH ($CXCR5^{hi}PD\text{-}1^{hi}$) cells, and expression of Ki-67 and BCL-6. **(B)** Ex vivo identification of macaque cTFH, T_{CM} and T_{EM} in PBMC. **(C)** Identification of OX-$40^{+}CD25^{+}$ cTFH following in vitro peptide pool or SEB stimulation. Gating corresponds to data presented in Figure 3F-I.

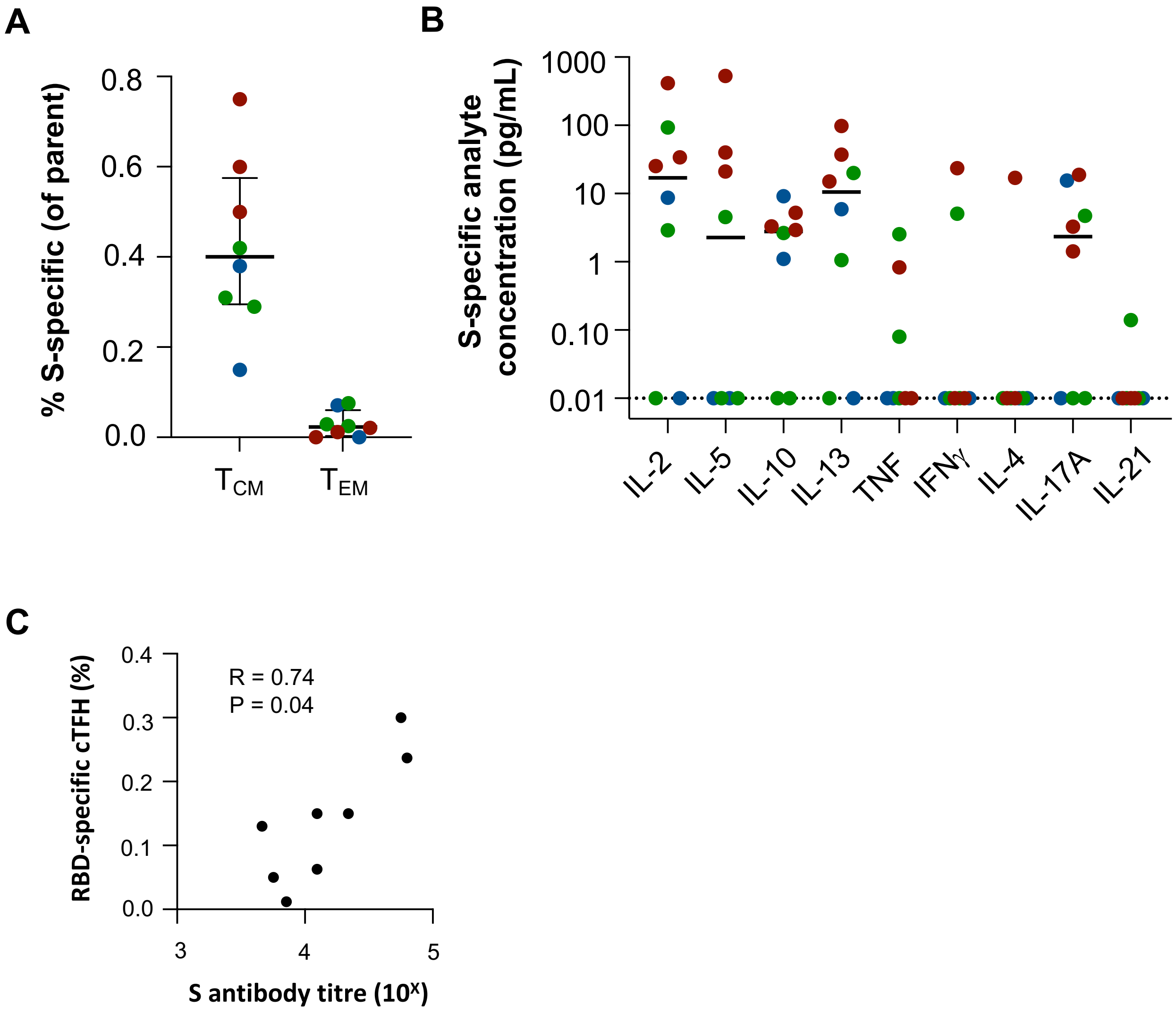

Supplemental Figure 13. S-specific T cell memory and cytokine production from macaque PBMC. **(A)** Quantification of total S-specific responses among T_{CM} or T_{EM} populations (n=8). Red, Spike/Spike group; green, Spike/RBD group; blue, RBD/RBD group. Data is presented as median ± IQR. (**B**) Quantification of cell culture supernatant cytokine concentrations following 18 hour stimulation of PBMC with overlapping peptides covering S. Line indicates median. **(C)** Spearman correlation of RBD-specific cTFH frequency and S antibody titres at D41.

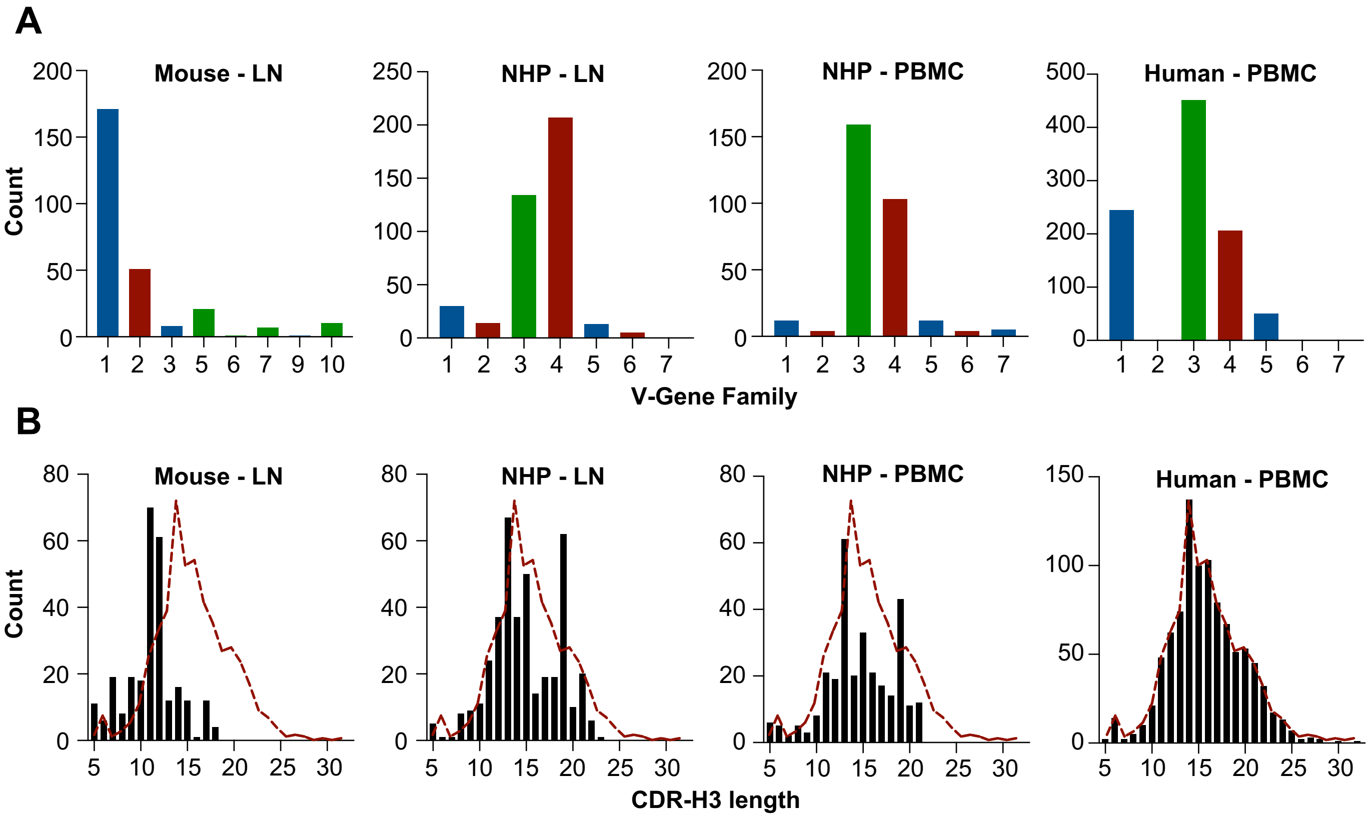

Supplemental Figure 14. (**A**) Distribution of V-gene family utilisation (colours indicate germline family) or (**B**) CDHR-H3 lengths of B cell receptor sequences recovered from: GC B cells ($B220^{+}IgD^{-}GL7^{+}$) within the draining iliac LN of C57BL/6 mice (n=3) 14 days after immunisation with S; RBD- and S-specific B cells ($CD20^{+}IgD^{-}IgG^{+}$) in the the draining iliac LN or PBMC of a single macaque 14 days after a second immunisation with S; or RBD- and S-specific B cells ($CD19^{+}IgD^{-}IgG^{+}$) within PBMC of convalescent COVID-19 subjects (n=6; as reported in Juno et al., 2020)

Mouse primers

Name	Sequence
1mFH_I	AGGAACTGCAGGTGTCC
1mFH_II	CAGCTACAGGTGTCCACTCC
1mFH_III	TGGCAGCARCAGCTACAGG
1mFH_IV	CTGCCTGGTGACATTCCCA
1mFH_V	CCAAGCTGTGTCCTGTC
1mFH_VI	TTTTAAAAGGTGTCCAGKGT
1mFH_VII	CCTGTCAGTAACTRCAGGTGTCC
1mFH_VIII	TTTTAAAAGGGGTCCAGTGT
1mFH_IX	CGTTCCTGGTATCCTGTCT
1mFH_X	ATGAAGTTGTGGYTRAACTGG
1mFH_XI	TGTTGGGGCTKAAGTGGG
1mRG* (Gamma)	AGAAGGTGTGCACACCGCTGGAC
2mFG*	GGGAATTCGAGGTGCAGCTGCAGGAGTCTGG
2mRG* (Gamma)	GCTCAGGGAARTAGCCCTTGAC

Non-human primate primers

Name	Sequence
RhH1-O	ATGGACTKGACCTGGAGG
RhH2-O	ATGGACTTGACCTGGAAG
RhH3-O	ATGGACTGGACCTGGAG
RhH4-O	ATGGACACGCTTTGCTCC
RhH5-O	ATGGAGTTTGGGCTGAGC
RhH6-O	ATGGAGTTTGGACTGAGC
RhH7-O	ATGGAGTTGGGACTGAGC
RhH8-O	ATGGAGTCGTGGCTGAG
RhH9-O	ATGGAGTTGGGGCTGAG
RhH10-O	GGAATTTAGGCTGAGCTG
RhH11-O	ATGGAATTTGGGCTGAGC
RhH12-O	GAAACACCTGTGGTTCTT
RhH13-O	ATGAAGCACCTGTGGGTC
RhH14-O	ATGAAGCACCTGTGGTTC
RhH15-O	ATGGGGTCAACTGCCATC
RhH16-O	ATGGAGTTKGGGCTGAGC
RhH17-O	ATGGAGTTTGKRCTGAGC
RhH18-O	ATGGAGTCRTGGCTGAGC
RhH19-O	ATGGAGTTTGTGCTGAGT
RhH20-O	ATGGGGTCCACCGTCAC
RhH21-O	ATGTCTGTCTCCTTCCTC
3CgCH1	GGAAGGTGTGCACGCCGCTGGTC
3RhCgCH1	AGGTGTGCACGCCGCTGG
RhH1-I	GCCCAGTCCCAGGTCCAG
RhH2-I	GTCCTGTCACAGGTGCAGCTG
RhH3-I	GCCCACTCCCAGGTGCAG
RhH4-I	GTCCAGTCCCAGGTCCAGC
RhH5-I	GCCCACTCCCTGGTGCAG
RhH6-I	GCCCACTCCGAGGTCCAG
RhH7-I	CGCCCACTCTGAGGTCCAG
RhH8-I	GGTGTCCAGTCCCAAGTCCAAC
RhH9-I	GGTCCTGTCCCAGGTGCAG
RhH10-I	GGGTCCTGTCCCAGGTGAAG
RhH11-I	TGTCCTGTCACAGGTGCAGC
RhH12-I	GTCCTGTCCCTGGTGCAGC
RhH13-I	GTCCTGTCCCTGGTGCAGC
RhH14-I	GTCCTGTCCCAGCTGCAGC
RhH15-I	GGGTCTTGTCCCAGGTGCAG
RhH16-I	GGTGTCCTGTCACAGGTGCAG
RhH17-I	GGGTCCTGTCACAGGTGCAG
RhH18-I	GGGTCTTGTCCCAGGTGCAG
RhH19-I	GGGTAGTGTCCCAGGTGCAA
RhH20-I	GGCCTGTCCCAGGTGCAG
RhH21-I	GGGTCTTGTCCCAGGTGACC
RhH22-I	GGCTCTTGTCCCAGGTGACC
RhH23-I	GGGTCTTGTCCCAGGTGACC
RhH24-I	GGGTCCTGTCCCAGGTACAG
RhH25-I	GGGTCCTGTCCCAGGTACAAC
RhH26-I	GGTCCTGTCCCAGGTGCAC
RhH27-I	GGTCCTGTCCCAGGTGCAA
RhH28-I	GAGTCTGTGCCGAGGTGCAG
RhH29-I	GGTGTCCAGTGTGACGTGCAG
RhH30-I	GGTGTCCAGTGTGAAGTGCAG
RhH31-I	GGTGTCCAGTGTGAGGTGCAG
RhH32-I	GGTGTCCAGTGTGAGGTGCAA
RhH33-I	GGTGTCCAGTGTGAGATGCAGC
RhH34-I	GGTGTCCAGTGTGAGGTGCA
RhH35-I	GGTGTCCAGTGTGAGGTGCG
RhH36-I	GGTGTCCAGTGTGAGGTGAAGTTG
RhH37-I	GGGTCTTGTCCCAGATGCAGC
RhH38-I	GGTCCTGTCCCAGCTGCAG
RhH39-I	TCCCAGTGTGAGGTGCAGC
3IgGInt	GTTCGGGGAAGTAGTCCTTGAC

Supplementary Table 1. Primers for B cell receptor heavy chain sequencing
