# Supplementary material for: Immunogenicity of prime-boost protein subunit vaccine strategies against SARS-CoV-2 in mice and macaques
Source: Nat Commun. 2021 Mar 3;12:1403. doi: 10.1038/s41467-021-21665-8 (PMC7930087; doi:10.1038/s41467-021-21665-8)
Supplement: Supplementary file 2 — Reporting Summary [file 41467_2021_21665_MOESM2_ESM.pdf]

nature research

Corresponding author(s): Wheatley, AK and Kent SJ

Last updated by author(s): Feb 2, 2021

Reporting Summary

Nature Research wishes to improve the reproducibility of the work that we publish. This form provides structure for consistency and transparency in reporting. For further information on Nature Research policies, see our Editorial Policies and the Editorial Policy Checklist.

Statistics

For all statistical analyses, confirm that the following items are present in the figure legend, table legend, main text, or Methods section.

n/a	Confirmed	
☐	☒	The exact sample size (*n*) for each experimental group/condition, given as a discrete number and unit of measurement
☐	☒	A statement on whether measurements were taken from distinct samples or whether the same sample was measured repeatedly
☐	☒	The statistical test(s) used AND whether they are one- or two-sided<br>*Only common tests should be described solely by name; describe more complex techniques in the Methods section.*
☒	☐	A description of all covariates tested
☒	☐	A description of any assumptions or corrections, such as tests of normality and adjustment for multiple comparisons
☐	☒	A full description of the statistical parameters including central tendency (e.g. means) or other basic estimates (e.g. regression coefficient) AND variation (e.g. standard deviation) or associated estimates of uncertainty (e.g. confidence intervals)
☐	☒	For null hypothesis testing, the test statistic (e.g. *F*, *t*, *r*) with confidence intervals, effect sizes, degrees of freedom and *P* value noted<br>*Give P values as exact values whenever suitable.*
☒	☐	For Bayesian analysis, information on the choice of priors and Markov chain Monte Carlo settings
☒	☐	For hierarchical and complex designs, identification of the appropriate level for tests and full reporting of outcomes
☒	☐	Estimates of effect sizes (e.g. Cohen's *d*, Pearson's *r*), indicating how they were calculated

Our web collection on statistics for biologists contains articles on many of the points above.

Software and code

Policy information about availability of computer code

Data collection	Flow cytometry data were collected in BD FACS Diva.
Data analysis	Data were analysed in Prism 8.0, r version 3.6.3, Legendplex analysis software v8.0, FlowJo v9 or 10, and IMGT V-quest 3.5.19

For manuscripts utilizing custom algorithms or software that are central to the research but not yet described in published literature, software must be made available to editors and reviewers. We strongly encourage code deposition in a community repository (e.g. GitHub). See the Nature Research guidelines for submitting code & software for further information.

Data

Policy information about availability of data

All manuscripts must include a data availability statement. This statement should provide the following information, where applicable:

- Accession codes, unique identifiers, or web links for publicly available datasets
- A list of figures that have associated raw data
- A description of any restrictions on data availability

The data that support the findings of this study are available in the Source Data File provided with this paper, and are otherwise available from the corresponding author upon reasonable request.

Field-specific reporting

Life sciences study design

All studies must disclose on these points even when the disclosure is negative.

Sample size	The mouse experiments were appropriately powered based on previous serological trials (as in Tan et al JCI 2019 doi: 10.1172/JCI123366). Macaque experiments were based on the number of animals available.
Data exclusions	No data were excluded from any experiments
Replication	Mouse experiments were successfully replicated in two independent experiments (i.e. a sample size of 10 represents two experiments of n=5). Due to limitations in large animal trials, non-human primate experiments were not replicated.
Randomization	All animals were randomly allocated into vaccine groups.
Blinding	Investigators were blinded to the immunisation status of the macaques during data acquisition and analysis. For mouse experiments, investigators were not blinded since all data was batch analysed according to the same gating schemes.

Reporting for specific materials, systems and methods

We require information from authors about some types of materials, experimental systems and methods used in many studies. Here, indicate whether each material, system or method listed is relevant to your study. If you are not sure if a list item applies to your research, read the appropriate section before selecting a response.

Materials & experimental systems

n/a	Involved in the study	
☐	☒	Antibodies
☐	☒	Eukaryotic cell lines
☒	☐	Palaeontology and archaeology
☐	☒	Animals and other organisms
☐	☒	Human research participants
☒	☐	Clinical data
☒	☐	Dual use research of concern

Methods

n/a	Involved in the study	
☒	☐	ChIP-seq
☐	☒	Flow cytometry
☒	☐	MRI-based neuroimaging

Antibodies

Antibodies used	For ex vivo TFH quantification from mice, freshly isolated LN ; single cell suspensions were stained with the following antibodies: Live/dead Red (Life Technologies), CD3 BV510 (145-2C11; Biolegend), PD-1 BV786 (29F.1A12; Biolegend), CXCR5 BV421 (L138D7; Biolegend), CD4 BUV737 (RM4-5; BD), B220 BV605 (RA3-6B2; BD), and F4/80 PE-Dazzle 594 (T45-2342; BD). Cells were permeabilized with transcription factor staining buffer (BD Biosciences) and stained intracellularly with anti-BCL-6 Alexa647 (IG191E/A8; Biolegend). Non-human primate LN suspensions and PBMC were stained with the same protocol, using the following antibodies: Live/dead Aqua (Life Technologies), CD3 Alexa700 (SP34-2; BD), PD-1 BV421 (EH12.2H7; Biolegend), CXCR5 PE (MU5UBEE; ThermoFisher), CD4 BV605 (L200; BD), CD20 BV510 (2H7; BD), CD8 BV650 (RPA-T8; Biolegend), CD95 BUV737 (DX2; BD), ICOS PerCP-Cy5.5 (C398.4A; Biolegend), CD69 FITC (FN50; Biolegend), CCR6 BV785 (G034E3; Biolegend), CXCR3 Pe-Dazzle594 (G02H57; Biolegend), BCL-6 APC (IG191E/A8; Biolegend) and Ki67 BUV395 (B56; BD).<br>For antigen-specific TFH quantification, mouse cells were stained with CD154 APC-Cy7 (TRAP1; BD), viability dye (Red or Aqua, Life Technologies), CD3 BV510 (145-2C11; Biolegend), CD25 BB515 (PD61; BD), 528 PD-1 BV786 (29F.1A12; Biolegend), CXCR5 BV421 (L138D7; Biolegend), CD4 BUV737 (RM4-5; BD), OX-40 PeCy7 (OX-86; Biolegend), B220 BV605 (RA3-6B2; BD), and F4/80 PE-Dazzle 594 (T45-2342; BD). NHP cells were stained with the following antibodies: CD3 Alexa700 (SP34-2; BD), PD-1 BV421 (EH12.2H7; Biolegend), CXCR5 PE (MU5UBEE; ThermoFisher), CD4 BV605 (L200; BD), CD20 BV510 (2H7; BD), CD8 BV650 (RPA-T8; Biolegend), CD95 BUV737 (DX2; BD), CCR6 BV785 (G034E3; Biolegend), CXCR3 Pe-Dazzle594 (G02H57; Biolegend), CD25 APC(BC96; Biolegend), and OX-40 BUV395 (L106; BD).<br>For mouse B cell analysis, cells were stained with the following antibodies: B220 BUV737 (RA3-6B2), IgD BUV395 (11-26c.2a), CD45 Cy7APC (30-F11), SA BV786 (BD), GL7 Alexa488 (GL7), CD38 Cy7PE (90), CD3 BV786 (145-2C11) and F4/80 BV786 (BM) (Biolegend).<br>Macaque B cells were stained with: IgD AF488 (polyclonal; Southern Biotech), IgM BUV395 (G20-127), IgG BV786 (G18-145) (BD), CD14 BV510 (M5E2), CD3 BV510 (OKT3), CD8a BV510 (RPA-T8), CD16 BV510 (3G8), CD10 BV510 (HI10a), CD20 APC-Cy7 (2H7) (Biolegend) and SA BV510 (BD), BCL-6 PE-Cy7 (K112-91, BD) and Ki-67 BUV395 (B56, BD).<br>For RBD/ACE2 and IgG ELISAs, the following antibodies were used: Pierce™ High Sensitivity Streptavidin-HRP (ThermoFisher Scientific Cat no: 21130); IgG, Rabbit Anti-Human, Polyclonal, HRP Ig fraction, 2 mL (Dako Agilent Cat: P021402-2); Anti-IgG-HRP, Macaque Pan-Species [1B3-HRP] Antibody, 100ug (Kerafast Cat: ET0002); Anti-mouse IgG H+L Peroxidase conjugated (KPL/Serocare Cat 474-1806)
Validation	All antibodies used for murine cells are commercially validated on the manufacturer's website as flow cytometry reagents for mouse experiments. Antibodies used for macaque cells were commercial antibodies either raised against macaque antigens or validated to cross-react with macaque antigens according to the manufacturer's website.

Eukaryotic cell lines

Policy information about cell lines

Cell line source(s)	Vero cells were obtained from ATCC; Expi293F cells were obtained from ThermoFisher (#A14527).
Authentication	None of the cell lines used were authenticated.
Mycoplasma contamination	Vero cells tested mycoplasma negative. Expi293 cells were not tested for mycoplasma.
Commonly misidentified lines (See ICLAC register)	There are no commonly misidentified lines.

Animals and other organisms

Policy information about studies involving animals; ARRIVE guidelines recommended for reporting animal research

Laboratory animals	Female C57BL/6 or BALB/c mouse strains (8-12 weeks of age at time of experimental initiation). Animals were maintained on a 12hr light/dark cycle at 18-24 degrees C and minimum humidity of 40%. For non-human primates, eight male pig-tail macaques (Macaca nemestrina) (6-15 years old).
Wild animals	No wild animals were used in this study.
Field-collected samples	No field-collected samples were used in this study.
Ethics oversight	Animal studies and related experimental procedures were approved by the University of Melbourne Animal Ethics Committee (#1714193, #1914874). Macaque studies and related experimental procedures were approved by the Monash University Animal Ethics Committee (#23997).

Note that full information on the approval of the study protocol must also be provided in the manuscript.

Human research participants

Policy information about studies involving human research participants

Population characteristics	The human convalescent cohort was median 56 years old and 42% female. The characteristics are reported in Table 1.
Recruitment	Participants were recruited by contact with the study investigators, and through word of mouth among SARS-CoV-2 infection clusters. Participants may have self-selected for people with a greater interest in COVID-19 infection, but this is unlikely to affect the results of the study.
Ethics oversight	Human clinical study protocols were approved by the University of Melbourne Human Research Ethics Committee (#2056689), and all associated procedures were carried out in accordance with approved guidelines.

Note that full information on the approval of the study protocol must also be provided in the manuscript.

Flow Cytometry

Plots

Confirm that:

- [x] The axis labels state the marker and fluorochrome used (e.g. CD4-FITC).
- [x] The axis scales are clearly visible. Include numbers along axes only for bottom left plot of group (a 'group' is an analysis of identical markers).
- [x] All plots are contour plots with outliers or pseudocolor plots.
- [x] A numerical value for number of cells or percentage (with statistics) is provided.

Methodology

Sample preparation	PBMC were isolated from whole blood, and lymph node suspensions were prepared from whole lymph nodes. Samples were either stained upon thawing or fresh after isolation. For antigen-specific T cell assays, cells were stained after stimulation.
Instrument	Samples were acquired on a BD LSR Fortessa, or sorted on a BD FACS Aria III
Software	Samples were acquired using BD FACS Diva, and analysed using FlowJo version 9 or 10
Cell population abundance	B cells were single-cell sorted for BCR sequencing, so purity is not relevant
Gating strategy	Identification of bulk and antigen-specific mouse germinal centre B cells: (A) Lymphocytes were identified by FSC-A vs SSC-A gating, followed by doublet exclusion (FSC-A vs FSC-H). Live and CD3-F4/80-streptavidin- (dump channel) cells were gated

and CD45+B220+IgD- B cells identified. Germinal centre (GL7+CD38lo) B cells were then assessed for binding to SARS-CoV-2 spike (S) and/or SARS-CoV-2 RBD probes. (B) Frequencies of germinal centre B cells was alternatively measured based on BCL-6 expression in live, B220+CD3-CD4-F4/80- lymphocytes.

Identifiation of mouse TFH cells: Lymphocytes were identified by FSC-A vs SSC-A gating, followed by doublet exclusion (FSC-A vs FSC-H). Live and B220-F4/80-streptavidin- (dump channel) cells were gated. CD3+CD4+ T cells were identified, and TFH were defined as CXCR5++BCL6+.

Macaque B cell gating strategy: Lymphocytes were identified by FSC-A vs SSC-A gating, followed by doublet exclusion (FSC-A vs FSC-H), and gating on dump- (CD3-CD8-CD14-CD10-CD16-streptavidin-) live CD20+ B cells. (A) Antigen-specific germinal center B cells were identified from class-switched IgD- B cells and intracellular expression of BCL6 and Ki-67. Alternatively, (B) circulating memory B cells in PBMC samples were identified as CD20+IgD-. Antigen specificity was determined by binding to SARS-CoV-2 spike (S) and/or SARS-CoV-2 RBD probes.

Macaque T cell gating strategy: Lymphocytes were identified by FSC-A vs SSC-A gating, followed by doublet exclusion (FSC-A vs FSC-H), dump exclusion (dead, CD20+), and gating on CD3+ cells. CD4+CD8- T cells were gated, and TFH identified as CXCR5++PD-1++.

☒ Tick this box to confirm that a figure exemplifying the gating strategy is provided in the Supplementary Information.
